# MOFGPT: Generative Design of Metal–Organic Frameworks using Language Models

**DOI:** 10.1021/acs.jcim.5c01625

**Published:** 2025-08-28

**Authors:** Srivathsan Badrinarayanan, Rishikesh Magar, Akshay Antony, Radheesh Sharma Meda, Amir Barati Farimani

**Affiliations:** † Department of Chemical Engineering, 6612 Carnegie Mellon University, Pittsburgh, Pennsylvania 15213, United States; ‡ Department of Mechanical Engineering, 6612 Carnegie Mellon University, Pittsburgh, Pennsylvania 15213, United States; ¶ Department of Biomedical Engineering, 6612 Carnegie Mellon University, Pittsburgh, Pennsylvania 15213, United States; § Machine Learning Department, 6612 Carnegie Mellon University, Pittsburgh, Pennsylvania 15213, United States

## Abstract

The discovery of Metal–Organic Frameworks (MOFs) with application-specific properties remains a central challenge in materials chemistry, owing to the immense size and complexity of their structural design space. Conventional computational screening techniques such as molecular simulations and density functional theory (DFT), while accurate, are computationally prohibitive at scale. Machine learning offers an exciting alternative by leveraging data-driven approaches to accelerate materials discovery. The complexity of MOFs, with their extended periodic structures and diverse topologies, creates both opportunities and challenges for generative modeling approaches. To address these challenges, we present a reinforcement learning-enhanced, transformer-based framework for the de novo design of MOFs. Central to our approach is MOFid, a chemically informed string representation encoding both connectivity and topology, enabling scalable generative modeling. Our pipeline comprises three components: (1) a generative GPT model trained on MOFid sequences, (2) MOFormer, a transformer-based property predictor, and (3) a reinforcement learning (RL) module that optimizes generated candidates via property-guided reward functions. By integrating property feedback into sequence generation, our method drives the model toward synthesizable, topologically valid MOFs with desired functional attributes. This work demonstrates the potential of large language models, when coupled with reinforcement learning, to accelerate inverse design in reticular chemistry and unlock new frontiers in computational MOF discovery.

## Graphical abstract

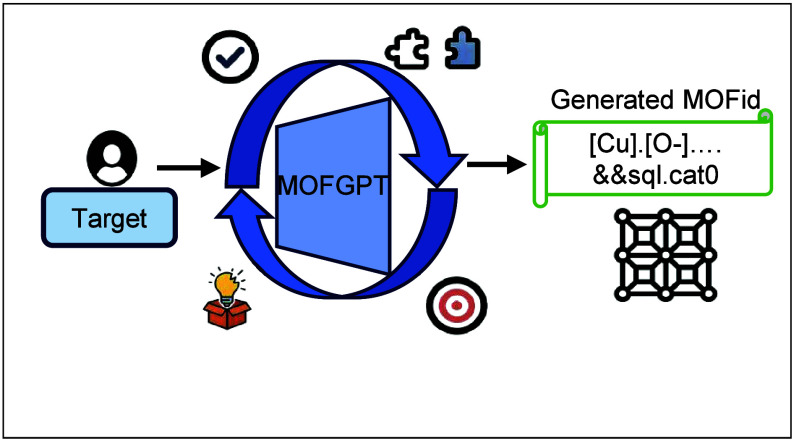

## Introduction

Metal–Organic Frameworks (MOFs) are porous crystalline materials consisting of inorganic metal clusters linked with organic ligands, known for their exceptional thermal stability, high porosity, and diverse applications in gas storage,^1,2^ water treatment,^3–5^ and catalysis.^6–8^ With over 100,000 reported MOFs^9^ and countless hypothetical structures,^10^ their vast chemical space offers tremendous opportunities for tailored material design but presents significant challenges in identifying optimal candidates for specific applications.^11^

Traditional computational screening approaches using molecular simulations and density functional theory (DFT)^12,13^ face scalability limitations due to their high computational cost.^14^ Data-driven methods^15–19^ and language modeling,^20–24^ have emerged as promising alternatives for large scale screening of MOFs.^25–29^ The strong performance of deep learning models in property prediction has motivated efforts to explore such models as possible generative tools to enable the design of novel structures that satisfy specific functional requirements.^30–34^ However, MOFs present unique challenges for generative models due to their complex structures, diverse topologies, and heterogeneous chemical compositions.^35^ Generative modeling approaches for molecular design have evolved over time,^36–44^ yet it remains challenging for MOFs due to the large number of atoms, diverse topologies and the multiple degrees of freedom involved in their design.^45–51^ These characteristics make coordinate-based or graph-based generative models difficult to scale and limit their ability to generalize across different constraints. In contrast, sequence-based representations of MOFs open the door to alternative modeling strategies that can bypass these structural complexities.

Transformer-based language models^52,53^ offer a compelling alternative for processing MOF representations as sequential data. Building on the effectiveness of GPT-like models for molecular design using SMILES-based representations,^54^ we adopt specialized approaches to capture the characteristic features of MOFs. For the same, we leverage MOFid^55^ - a string-based encoding that captures both chemical composition through SMILES^56^ and topology through standardized RCSR labels.^57^ However, generative modeling alone cannot guarantee that MOF candidates possess desirable properties. Methods like supervised fine-tuning, though proven efficient at generating structures, are not guaranteed to be target-specific.^58^ Drawing inspiration from the success of reinforcement learning in optimizing large language models for specific tasks,^59^ we adapt these principles to MOF generation, enabling targeted design of structures with desired properties. Reinforcement learning (RL) has proven effective in molecular design,^60–63^ and we seek to adapt and replicate this success for MOFs, where both local chemistry and global topology must be optimized simultaneously.^64^ Building on this foundation, we introduce a comprehensive RL framework that enables multiparameter optimization, allowing for the simultaneous control of key structural and functional properties during generation.

In this work, we introduce a reinforcement learning-enhanced GPT-based generative framework for target-specific de novo MOF design as an alternative to existing supervised fine-tuning methods, as seen in Figure 1. Our RL approach integrates three key components: (1) a GPT-based MOF generator trained on MOFid sequences, (2) a transformer-based property predictor based on MOFormer,^20^ and (3) a reward policy gradient-based RL mechanism that optimizes generated MOFs toward targeted properties. Our framework rewards novelty, validity, and structural diversity while guiding generation toward specific property targets, overcoming limitations of purely generative approaches not having property-based evaluation.

**Figure 1:**
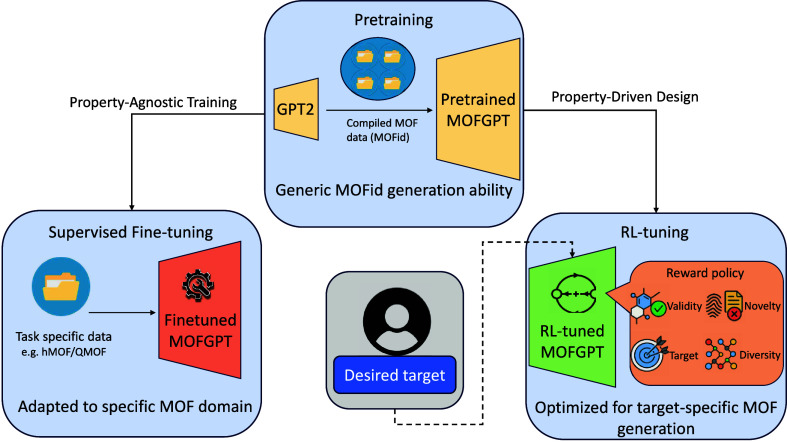
Overview of the MOFGPT framework. The pretrained MOFGPT model is first trained on a large, diverse corpus of MOF structures represented as MOFid strings to learn general MOF generation capabilities. This model is then fine-tuned using task-specific data sets (e.g., hMOF, QMOF) to adapt to particular domains or target properties. Alternatively, reinforcement learning (RL) is applied using a reward policy that optimizes for target-specific MOF generation with high validity, novelty, and diversity.

While prior work has explored reinforcement learning with building block-based MOF representations,^64^ our framework introduces a modular, property-driven generator trained on MOFid strings, leveraging a fixed property predictor (MOFormer) and multi-objective reward optimization. This combination enables structure generation directly at the MOFid level, bypassing manual assembly of topologies and fragments. Unlike other previous efforts that focused on database searches, rule-based design, or even those that use a generic GPT framework for materials generation,^65,66^ our approach provides a target-specific framework for MOF discovery with domain-adapted architecture trained specifically on MOFs. By combining transformer-based generative modeling with RL and property-aware feedback, we present an advance in AI-driven MOF discovery, enabling scalable, targeted exploration of the vast MOF design space.

## Methods

Our approach addresses the fundamental challenge of generating chemically valid MOFs with targeted properties through a three-stage computational pipeline that improves progressively: pretraining for general MOF generation capabilities, supervised fine-tuning (on property prediction) which guides MOF generation, and reinforcement learning for property-targeted optimization. This hierarchical approach ensures that the model first learns the basic ”grammar” of the MOF structures through pretraining, before being guided toward specific functional objectives. A salient feature of our framework is the integration of reinforcement learning with transformer-based generative models for property-targeted MOF design. The novelty lies in our comprehensive RL setup that provides explicit control over functional objectives while maintaining chemical validity and structural diversity through a multicomponent reward architecture specifically designed for MOF generation.

### Data Representation

A critical component of our approach is the efficient representation of MOF structures in a format suitable for language models. We utilize the MOFid representation,^55^ which encodes both chemical composition and topology in a compact string format. MOFid consists of two primary components: the SMILES notation of secondary building units (SBUs)^56^ and topology codes from the Reticular Chemistry Structure Resource (RCSR) database,^57^ connected by a separator token (“*&* *&*”):

MOFid=[organic_components]·[inorganic−components]&&[topology]·[catenation] 1

This representation enables our model to generate complete MOF descriptions including both building blocks and topology in a single integrated framework. It is important to note here that MOFids were standardized by ordering organic before inorganic SMILES strings using a consistent heuristic: organic SBUs were ranked by atom count and alphabetic SMILES sort. Non-canonical SMILES were canonicalized using RDKit. Only MOFs with resolvable RCSR topologies were retained; unresolved or ambiguous cases were excluded. We implemented a specialized tokenizer that processes both chemical and topological components, with all sequences standardized to a maximum length of 512 tokens. Further details on tokenization and representation can be found in the Supporting Information.

Although MOFid provides a convenient string-based representation for generative modeling, it represents a static view of MOF structures that assumes rigid frameworks and perfect crystallinity. While this enables efficient computational generation and property prediction, dynamic effects such as framework flexibility and defect chemistry are not captured in this representation. Our generated MOFs are chemically valid and topologically consistent, with predicted properties serving as useful estimates for identifying promising candidates, though experimental validation remains important for confirming performance in real applications.

Our study aggregates MOF structures from three comprehensive repositories: Boyd and Woo,^2^ quantum MOF (QMOF),^67,68^ and hypothetical MOF (hMOF)^10^ data sets, creating a training set of 323,469 samples and a held-out test set of 81,260 samples. This combined data set is used to pretrain our GPT framework. While newer databases such as 2D EC-MOF^69^ for electrochemical applications and ToBaCCo^70^ for hypothetical structures have emerged, we focused on the established Boyd and Woo, hMOF, and QMOF data sets to maintain consistency with the frozen MOFormer property predictor’s training data and avoid distribution mismatch between generator and predictor components.

On top of the pretraining data set, we have task-specific data sets for our fine-tuning and reinforcement learning approaches. For the same, we leverage two distinct property data sets: (1) the hMOF data set for gas adsorption properties, which consists of gas adsorption values (in mol/kg) for CO_2_ and CH_4_ at various pressures, and (2) the QMOF data set for electronic band gap values (in eV), thereby providing diverse objective functions for our optimization framework. For an unbiased study, all the task-specific hMOF data sets have the same set of MOFids subject to adsorption of the two gases at different pressures. In our study specifically, we utilize 5 CH_4_ adsorption data sets at pressures of 0.05, 0.5, 0.9, 1.0, and 5.0 bar, 5 CO_2_ adsorption data sets at pressures of 0.01, 0.1, 0.5, 1.0, and 5.0 bar, and 1 electronic band gap data set from QMOF. For our primary results demonstration, we selected representative conditions to showcase the framework’s versatility across different physical regimes and property types. Additional data sets are utilized in supplementary analyses to validate the generalizability of our approach across the full range of adsorption conditions.

The majority of our training structures originate from the hMOF data set focused on gas adsorption properties, while electronic property data from QMOF represents a smaller subset (∼20,000 structures). However, our framework’s reliance on the frozen MOFormer predictor, which was trained and validated on electronic properties, helps mitigate potential limitations in this domain. The observed successful band gap optimization (as seen in Results - Table 1) suggests that the combination of general MOF structural knowledge from pretraining and specialized electronic property prediction from MOFormer provides sufficient capability for this task.

For gas adsorption properties, higher values indicate better performance; therefore the optimization objective is to predict materials with property values greater than or equal to the desired target, since increased uptake capacity is desirable for applications such as gas storage and separation. For electronic band gap optimization, lower values are typically targeted to improve electrical conductivity, which is particularly valuable for energy storage and electronic device applications - so the optimization objective is to generate materials with band gap property values lower than or equal to the desired target.

### Model Architecture and Training Methodology

Our MOFGPT framework utilizes a transformer-based architecture derived from GPT-2, adapted specifically for processing MOFid representations. The model consists of 12 transformer decoder layers with an embedding dimension of 768, and 12 attention heads, with a feed-forward dimension of 3072. This configuration provides sufficient capacity to capture the complex relationships in MOF structures while remaining computationally tractable. The training methodology follows a three-stage approach progressively builds model capabilities:

#### Stage 1: Pretraining for General MOF Generation

We first pretrain the base language model using next-token prediction on our large corpus of MOF structures. The pretraining objective maximizes the log-likelihood of the next token given previous tokens:

$$L_{\mathrm{pretrain}}=-\overset{T}{\underset{t=1}{\sum }}\log P_{\theta }\left(x_{t}|x_{<t}\right)$$ 2

where *x*_*t*_ is the token at position *t*, *x*_<*t*_ represents all tokens before position *t*, and *P*_θ_ is the probability distribution over the vocabulary given by the model with parameters θ. This stage enables the model to learn the fundamental patterns and constraints of MOF structures, including chemical bonding rules, coordination environments, and topological relationships. The pretraining phase is crucial for establishing a strong foundation that ensures generated structures remain chemically sensible throughout subsequent optimization.

#### Stage 2: Supervised Fine-tuning for Property Prediction

After pretraining, we fine-tune the model for property prediction using supervised learning. We extend the base language model with a specialized regression head that processes the output embeddings from the transformer decoder through a series of feed-forward layers to extract property-relevant features. The fine-tuning objective minimizes the mean squared error between predicted and actual property values:

$$L_{\mathrm{fine‐tune}}=\frac{1}{N}\overset{N}{\underset{i=1}{\sum }}{\left(y_{i}-{ŷ}_{i}\right)}^{2}$$ 3

where *y*_*i*_ is the ground truth property value for the *i*th MOF, *ŷ*_*i*_ is the predicted value, and *N* is the number of training examples. This stage is essential for two reasons: first, it allows the model to capture structure–property relationships, and second, it creates a property predictor that will serve as the ”value function” in our reinforcement learning framework. The dual role of this fine-tuned model as both generator and property evaluator ensures consistency between generation and evaluation processes. Since fine-tuning does not guarantee target-specific generations and fails to produce valid structures in our implementation, we explore RL as a strong and compelling alternative for our objective. While other MOF generative models in the literature may achieve different results with fine-tuning approaches, our framework demonstrates that RL provides a robust solution to the fundamental challenge of generating both valid and property-targeted MOF structures.

#### Stage 3: Reinforcement Learning Framework

The reinforcement learning stage optimizes the pretrained model to generate MOFs with specific target properties. The RL framework consists of two main components: (1) Policy network, here the language model that generates possible outputs at each step, and (2) Value network, here the frozen property prediction model (MOFormer^20^) that is used to estimate the expected reward for a generated MOF sequence.

As illustrated in Figure 2, the RL training loop consists of four main steps: (1) generating a batch of MOF structures with the generative model, (2) evaluating each structure using the frozen property predictor, (3) calculating policy gradients based on computed rewards, and (4) updating policy parameters accordingly. The RL objective is to maximize the expected reward:

$$J\left(\theta \right)=E_{\tau ∼\pi_{\theta }}\left[R\left(\tau \right)\right]$$ 4

where π_θ_ is the policy defined by the model parameters θ and *R*(τ) is our multi-component reward function. The policy gradient update follows the REINFORCE with Baseline algorithm,^71^ which aims to maximize the expected reward by adjusting the policy parameters. The gradient of the expected reward *J*(θ) with respect to the policy parameters θ is approximated as

$$\nabla_{\theta }J\left(\theta \right)\approx \frac{1}{N}\overset{N}{\underset{i=1}{\sum }}\overset{T_{i}}{\underset{t=1}{\sum }}\nabla_{\theta }\;\log\pi_{\theta }\left(a_{t}^{i}|s_{t}^{i}\right)\cdot \left(R_{i}-\mu \right)\cdot \gamma^{t-1}$$ 5

where π_θ_ is the policy, *a*_*t*_^*i*^ is the action (token) selected at time *t* for sequence *i*, *s*_*t*_^*i*^ is the corresponding state (partial sequence), *R*_*i*_ is the reward for the complete sequence, μ is the baseline (typically the mean reward), and γ is a discount factor.

**Figure 2:**
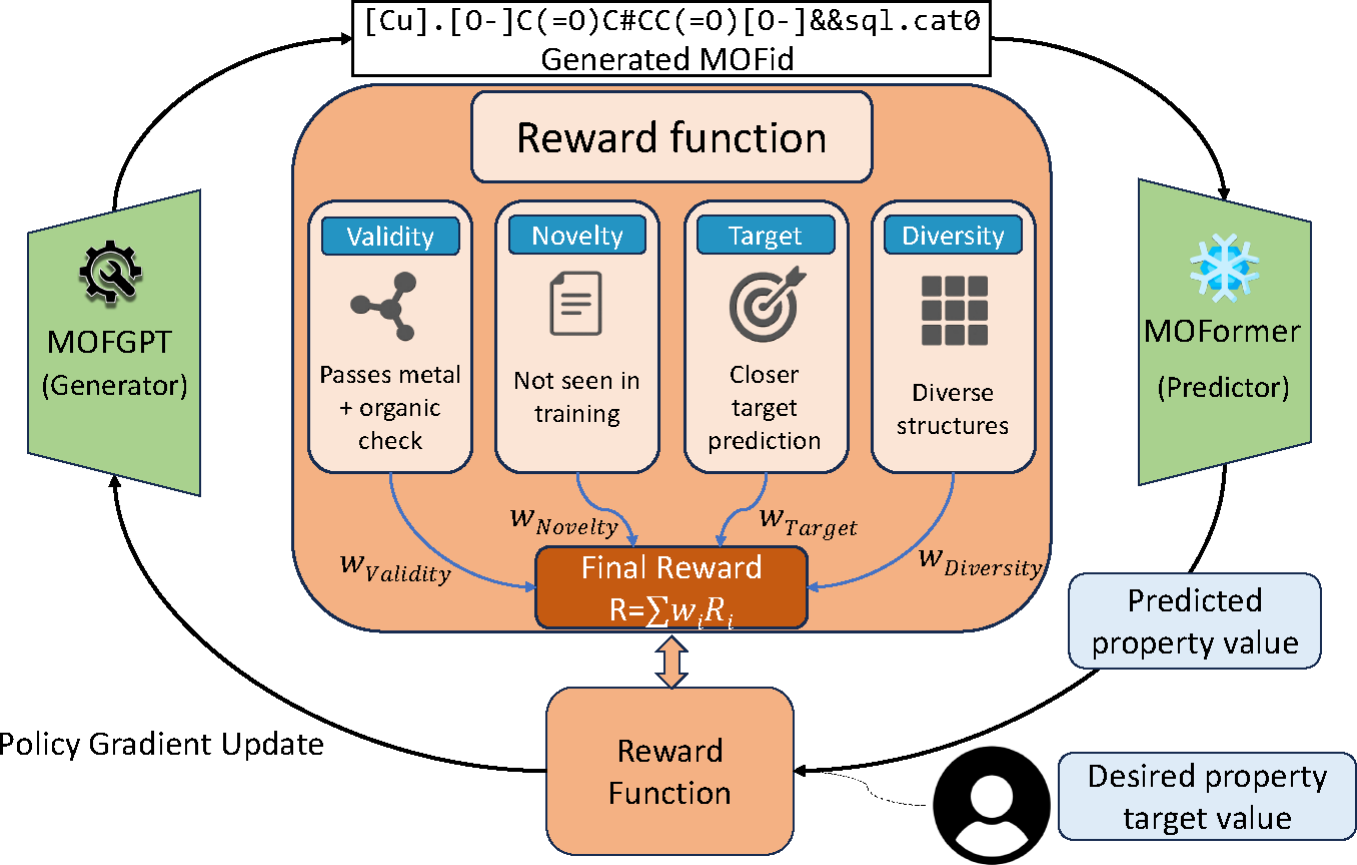
Reinforcement learning framework for property-driven MOF generation with multi-objective reward function. The RL-tuned MOFGPT model generates MOFid candidates evaluated by MOFormer. The reward function integrates four components: (1) validity (chemical and MOF-specific constraints), (2) novelty (penalizing training duplicates), (3) target accuracy (rewarding desired property values), and (4) diversity (encouraging structural variety). The weighted final reward R = ∑*w*_*i*_ *R*_*i*_ drives policy gradient updates to optimize the generation process.

It is important to note that the MOFormer model is used as a frozen property predictor throughout reinforcement learning. As it is not retrained in this work, we refer readers to the original MOFormer work^20^ for detailed benchmarking of its accuracy across gas adsorption and electronic properties. In our framework, MOFormer serves as a stable value estimator to guide property-conditioned generation.

The complete set of model configurations (for all three stages) and training hyperparameters are detailed in the Supporting Information.

### Reward Function Design

The effectiveness of our reinforcement learning approach depends critically on the reward function design. The design of our multi-component reward function addresses fundamental challenges unique to MOF generative modeling that distinguish it from small molecule generation. While small organic molecules can be validated through established chemical rules, MOFs require additional consideration of both local coordination chemistry and global topological consistency. This challenge necessitated our comprehensive reward architecture that balances multiple competing objectives while maintaining focus on property targeting. As illustrated in the center of Figure 2, our multicomponent reward function integrates four key objectives with carefully tuned weights:

$$R_{\mathrm{total}}\left(m\right)=\beta_{\mathrm{target}}\cdot R_{\mathrm{target}}\left(m\right)+\alpha_{n}\cdot R_{\mathrm{novelty}}\left(m\right)+\alpha_{v}\cdot R_{\mathrm{validity}}\left(m\right)+\alpha_{d}\cdot R_{\mathrm{diversity}}\left(m\right)$$ 6

where β_target_, α_*n*_, α_*v*_, and α_*d*_ are experimentally chosen weighting coefficients that balance property optimization, exploration, chemical feasibility, and structural variety, respectively (specific values provided in Supporting Information). These weights remain fixed throughout training to ensure consistent optimization objectives, though future work could explore adaptive weighting schemes for dynamic multi-objective optimization. Here, we acknowledge that optimal reward weights may vary across specific applications, and while our chosen configuration provides robust performance across diverse MOF optimization tasks, users can further tune these parameters for their particular data sets and targeting requirements.

The total reward calculation however follows an adaptive two-tier architecture that differentiates between promising and poor-performing structures. High-performing structures (selected via top-K mechanism explained later in this section) receive full multi-component rewards including target proximity, validity bonuses, novelty incentives, and diversity encouragement. Lower-performing structures receive reduced target signals to focus learning on promising candidates. This hybrid structure ensures target property optimization dominates the reward signal while maintaining exploration across the full chemical space. Additionally, our framework supports extension to multi-objective optimization via scalar-weighted reward terms, though dynamic weighting or curriculum RL may be needed for more complex trade-offs.

#### Core Reward Components

##### Target Property Reward

The target property reward guides generation toward structures with desired functional properties, serving as the primary optimization objective:

$$R_{\mathrm{target}}\left(m\right)=\overset{k}{\underset{i=1}{\sum }}w_{i}\cdot R_{\mathrm{proximity}}\left({\mathrm{p̂}}_{i}\left(m\right),T_{i}\right)$$ 7

where *p̂*_*i*_(*m*) is the predicted property value, *T*_*i*_ is the target value, and *w*_*i*_ is the importance weight. The proximity reward implements a tiered structure with distinct achievement levels: excellent performance (within 5% of target, reward = 15.0), very good (5–10%, reward = 12.0), good (10–20%, reward = 8.0), moderate (20–50%, reward = 4.0), and poor but improving performance with linearly decreasing rewards. This tiered reward structure addresses the challenge that simple distance-based metrics fail to distinguish between meaningful progress toward targets versus random fluctuations around poor performance levels. Additionally, we also provide direction-specific bonuses to address the asymmetric nature of property optimization where the desired direction varies by application. For gas adsorption properties, values meeting or exceeding targets indicate better performance, while for electronic properties like band gaps, values at or below targets are preferred for better conductivity.

##### Novelty Reward

The novelty reward encourages exploration of new chemical space by identifying structures not present in the training data:

$$R_{\mathrm{novelty}}\left(m\right)=\{\begin{array}{ll} 1 & \mathrm{if}\;m\notin D_{\mathrm{train}}\\ 0 & \mathrm{otherwise} \end{array}$$ 8

where $D_{\mathrm{train}}$ represents the set of MOF structures in the training data set. This binary reward prevents overfitting to known MOF structures while maintaining computational efficiency through exact string matching of MOFid representations.

##### Validity Reward

The validity reward ensures chemical feasibility by verifying that generated structures satisfy established chemical and topological constraints:

$$R_{\mathrm{validity}}\left(m\right)=\{\begin{array}{ll} 1 & \mathrm{if valid according to validation procedure}\\ 0 & \mathrm{otherwise} \end{array}$$ 9

Our validation procedure encompasses multiple checks: SMILES syntax validation using RDKit with metal atom substitution, metal node presence verification, structural balance confirmation requiring both organic and inorganic components, topology validity against known RCSR database entries when topology tokens are present, and coordination number validation for metal centers. It is important to note that, while validity assesses chemical feasibility, it does not guarantee synthetic accessibility or experimental constructability.

##### Diversity Reward

The diversity reward prevents mode collapse by encouraging structural variety within generated batches through multiple complementary metrics:

$$R_{\mathrm{diversity}}\left(m,B,H\right)=w_{b}\cdot S_{\mathrm{batch}}\left(m,B\right)+w_{n}\cdot S_{\mathrm{ngram}}\left(m,B\right)+w_{h}\cdot S_{\mathrm{history}}\left(m,H\right)+w_{c}\cdot S_{\mathrm{composition}}\left(m\right)$$ 10

where B is the current batch, H is the generation history, and the weights are *w*_*b*_ = 0.30 (batch diversity), *w*_*n*_ = 0.25 (n-gram pattern diversity), *w*_*h*_ = 0.35 (historical uniqueness), and *w*_*c*_ = 0.10 (compositional diversity). This multifaceted approach captures different aspects of structural variety to ensure broad exploration of the chemical space. As part of this reward, we maintain a rolling memory of 500 previously generated structures ( H ) that prevents cycling between high-reward candidates and ensures continued exploration of new chemical space throughout the optimization process.

The data sets used in our study (hMOF/QMOF) exhibit known compositional biases, particularly overrepresentation of Zn and Cu clusters. To mitigate potential mode collapse into these overrepresented chemistries when targeting extreme properties, our diversity reward component plays a crucial role. The diversity reward specifically includes compositional diversity (*S*_composition_) that encourages exploration beyond common metal-linker combinations. Additionally, the historical uniqueness component (*S*_history_) maintains a rolling memory of 500 previously generated structures, preventing cycling between high-reward but compositionally similar candidates. This architecture ensures that even when optimizing for extreme targets, the framework continues to explore less common metal nodes and novel linker chemistries rather than converging solely on the most frequent training examples.

#### Advanced Reward Mechanisms

Beyond the core components, our reward system incorporates several mechanisms to improve training stability and exploration:

##### Global Memory

To prevent loss of high-performing structures discovered during training, we maintain a global memory of the best discoveries across all epochs:

$$M_{\mathrm{global}}={\left\{\left(m_{j},p_{j},s_{j},r_{j}\right)\right\}}_{j=1}^{200}$$ 11

where each entry contains a MOF structure *m*_*j*_, predicted properties *p*_*j*_, target progress score *s*_*j*_, and total reward *r*_*j*_. The target progress score provides unified ranking across all objectives:

$$s_{j}=\overset{k}{\underset{i=1}{\sum }}w_{i}\times {\mathrm{Progress}}_{i}\left(p_{j,i},T_{i}\right)$$ 12

The progress function rewards both achievement and overachievement in the desired optimization direction, ensuring structures exceeding targets receive bonuses proportional to their performance while maintaining minimum scores for all candidates. This global memory ensures that promising structures discovered at any point during training are retained and can guide future generations.

##### Top-K Selection

Early in training, most generated structures are invalid or far from targets. Our top-K implementation focuses learning on promising structures by selecting only the highest-performing candidates. Our implementation also becomes increasingly selective as training progresses (50% early training, decreasing to 30% later). This progressive selectivity is established due to the model’s improving capability - early training requires broader sampling to establish basic chemical competency, while later training benefits from concentrated optimization of promising candidates.

##### Reward normalization

Reward normalization becomes critical, as unnormalized rewards can prevent effective policy updates. Our reward normalization implementation applies scaling only when batch statistics indicate extreme outliers or excessive variance, preserving natural reward relationships while preventing training instability.

These mechanisms work together to address key challenges in MOF generation: balancing exploration of new structures with focused optimization toward targets, maintaining consistent performance across different property ranges, and preventing mode collapse while still converging to high-quality solutions. The complete mathematical formulations, implementation details, and parameter values (chosen through experimentation) for all reward components are provided in the Supporting Information.

## Results and Discussion

### Fine-tuned Models Fail to Generate Valid MOFs

A critical finding that motivates our entire RL approach is that fine-tuned models without RL optimization fail to generate any chemically valid MOF structures across all property domains and targets. This failure to produce viable chemical structures represents a limitation of supervised fine-tuning approaches and establishes the necessity of our reinforcement learning framework.

The fine-tuned model, despite successful property prediction during training, cannot translate this predictive capability into generation of chemically feasible MOF structures. While the model produces numerical property values, these correspond to structurally invalid MOFs that violate basic chemical rules, coordination constraints, and topological requirements. This validity challenge underscores why property targeting in MOF generation requires sophisticated reward-guided optimization rather than simple fine-tuning approaches. This fundamental limitation motivated our reinforcement learning approach, which we demonstrate successfully addresses both validity and property targeting simultaneously.

It is important to note that our comparison is primarily against our own supervised fine-tuning baseline rather than other published MOF generative models. Direct comparison with other MOF-specific generative models is challenging due to differences in representation schemes (MOFid vs other encodings), data sets, and evaluation metrics. However, our framework’s ability to generate valid MOFs (35–100% validity rates depending on targets) while targeting specific properties represents a significant advancement over approaches that cannot produce chemically feasible structures, as demonstrated by our fine-tuned baseline’s 0% validity across all tested conditions.

### RL Framework: Simultaneous Validity and Property Control

Our results demonstrate three key achievements: resolution of the validity issues affecting generative MOF design, systematic property targeting across diverse domains, and achieving structural diversity during optimization. Here, we show results demonstrating that our reinforcement learning approach achieves chemical validity and property targeting across four distinct tasks: CH_4_ adsorption at 0.05 bar, CH_4_ adsorption at 0.9 bar, CO_2_ adsorption at 0.01 bar, and electronic band gap values. These pressure conditions were specifically chosen to represent distinct adsorption regimes with practical significance: low-pressure methane adsorption (0.05 bar) for testing fundamental selectivity limits, reduced-pressure methane conditions (0.9 bar) relevant to vacuum swing adsorption and depressurization scenarios, and dilute CO_2_ capture (0.01 bar) representing intermediate conditions between direct air capture and flue gas processing applications. Additional pressure conditions and extended results are provided in the Supporting Information. This selection spans both gas separation/storage and electronic property domains, demonstrating cross-domain applicability while targeting practically important operating conditions. For each task, we generated structures until obtaining 30 that satisfied all criteria (valid, novel, and diverse). While larger sample sizes can smooth distributions, our aim is to visualize directional shifts rather than estimate full population statistics. The choice of N = 30 also facilitates per-sample inspection, allowing structural and chemical trends to be traced more easily. The percentages reported in Table 1 represent the fraction of total generations meeting each criterion.

**Table 1:** Performance Metrics for RL-Based MOF Generation across Different Property Targets

**Property**	**Target**	**Validity (%)**	**Novelty (%)**	**Diversity (%)**
CH_4_ adsorption at 0.05 bar (mol/kg)	Mean	49.2	90.9	97
	Mean + 1σ	39.5	83.33	97
	Mean + 2σ	58.3	85.71	97
CH_4_ adsorption at 0.9 bar (mol/kg)	Mean	71.21	63.82	100
	Mean + 1σ	52	76.92	99
	Mean + 2σ	53.42	76.92	97
CO_2_ adsorption at 0.01 bar (mol/kg)	Mean	42.85	83.33	99
	Mean + 1σ	50	83.33	99
	Mean + 2σ	100	100	100
Band gap (eV)	Mean	100	93.75	93.75
	Mean + 1σ	64	93.75	100
	Mean + 2σ	35.63	100	83.0
	Mean – 1σ	39.75	90.90	100

We choose strategic targets for each particular data set based on the corresponding original data distributions: we analyze the mean and standard deviation (σ) of the original distributions and choose three targets each corresponding to mean, mean+σ and mean+2σ of the original data distributions. Additionally we choose mean-σ for band gap optimization, since a lower numeric value is a better target for that task. This systematic target selection strategy enables comprehensive evaluation of our framework’s performance across the full spectrum of difficulty levels: mean targets test basic property control, mean+σ targets (representing ∼ 84th percentile performance) evaluate moderate optimization capability, while mean+2σ targets (representing ∼ 97.5th percentile, or top 2.5% of materials) probe the framework’s ability to discover exceptional materials in the extreme tail of the property distribution. The mean+2σ targets are particularly valuable as they correspond to materials with performance characteristics that are rarely found in natural MOF databases, representing the type of high-performance candidates that traditional screening approaches would struggle to identify. Figure 3 demonstrates our framework’s ability to systematically reshape property distributions, inducing a distribution shift toward desired regions while maintaining chemical validity - a capability completely absent in fine-tuned approaches.

**Figure 3:**
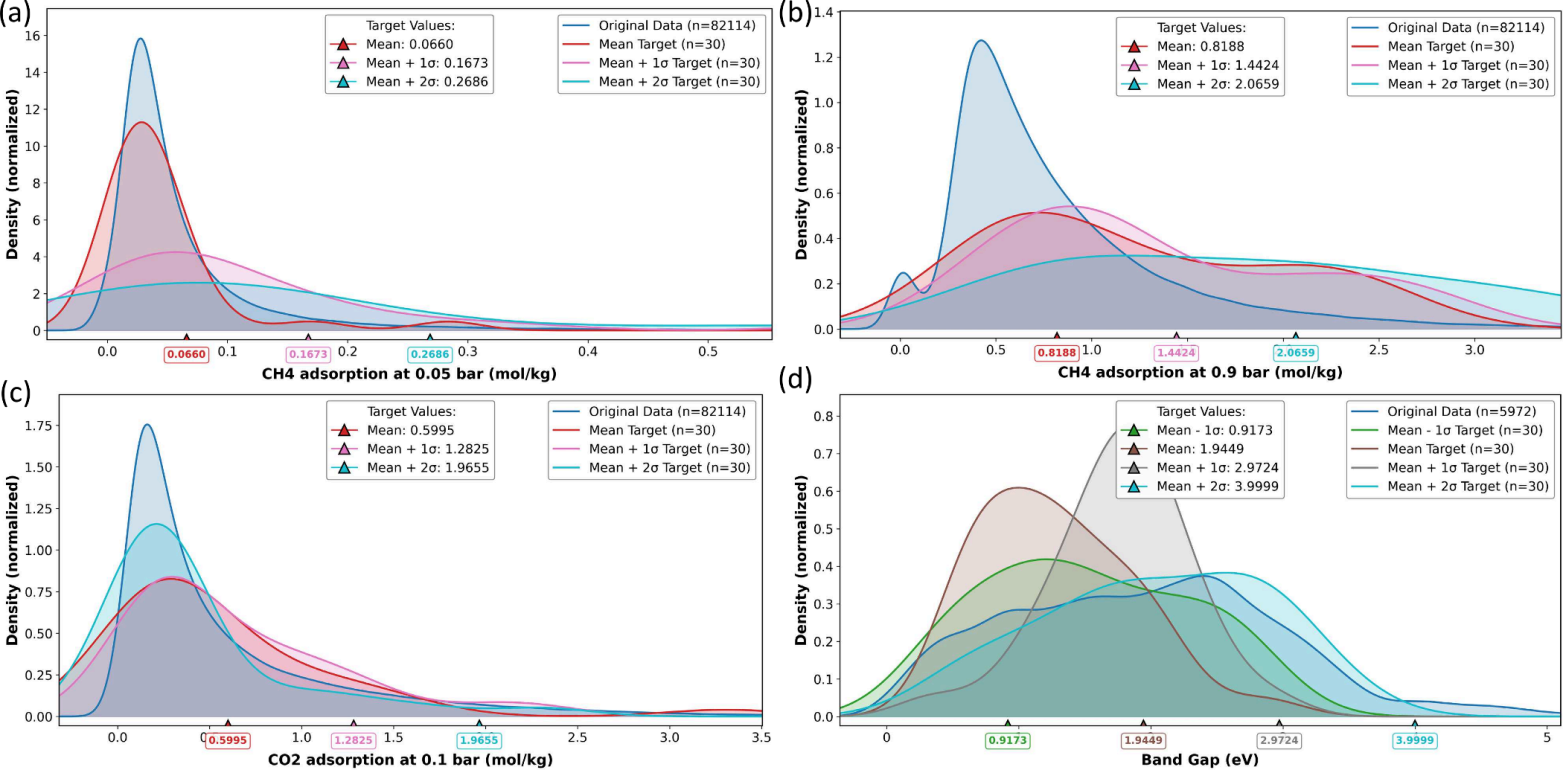
Property-targeted generation results across four tasks. (a) CH_4_ adsorption at 0.05 bar. (b) CH_4_ adsorption at 0.9 bar. (c) CO_2_ adsorption at 0.01 bar. (d) Electronic band gap optimization. RL-optimized distributions (colored curves) show focused targeting around desired property values, contrasting with the broad original data set distributions (blue).

Table 1 presents validation of our RL approach: achieving substantial validity rates while simultaneously optimizing for specific property targets. The stark contrast between invalid generations from fine-tuned models and the validity percentages for RL approaches across all scenarios demonstrates an advantage of our framework. We observe that even the most challenging extreme targets (mean+2σ) at the right tail of the original data distribution, which have very few naturally existing points, maintain substantial validity rates. This proves that chemical feasibility and ambitious property targeting are not mutually exclusive when proper reward-guided optimization is employed. However, we note that as MOFormer is not retrained, its predictions beyond +2σ of the training property distribution may be unreliable. We treat such predictions as heuristics for steering generation rather than absolute values, and note this limitation in high-target RL optimization.

Further, the validity metrics reveal trade-offs that reflect the realities of chemical space exploration. As optimization targets become more ambitious, validity rates show variation while novelty generally increases, indicating the framework is successfully exploring less-represented regions of chemical space where high-performance materials reside. For instance, for the CO_2_ adsorption data set, we attempt extreme targeting (mean+2σ), which achieves 100% validity, representing a remarkable accomplishment given that these targets correspond to the top 4.5% of property performance in the original data set. Similarly, band gap optimization maintains validity rates above 39% even for extreme targets (in this case the lower band gap values!), demonstrating the framework’s ability to navigate challenging regions of electronic property space while preserving chemical feasibility. Novelty rates are consistently more than 63% and diversity metrics consistently exceed 83% across all scenarios, demonstrating effective prevention of mode collapse even when optimizing for specific property targets.

Table 2 provides comprehensive statistical analysis of all chemically valid RL-generated structures. Beyond achieving specific means, our approach demonstrates potential control over entire distribution characteristics. For example, for CH_4_ adsorption at 0.05 bar, extreme targeting (mean+2σ) achieved a broader distribution (σ = 0.243 mol/kg) compared to the original data set variance (σ = 0.101 mol/kg), indicating successful exploration of high-performance regions. Band gap optimization shows distributional control across different targets, with standard deviations indicating the model centers reasonably around the desired targets. This tasks demonstrates versatility, spanning from better conductivity (lower band gaps) to improved insulating characteristics (higher band gaps).

**Table 2:** Statistical Properties of Generated Structures across Different Property Domains^a^

**Property**	**Data set (Target)**	**Mean**	**Std Dev**
CH_4_ adsorption at 0.05 bar (mol/kg)	Original Data	0.066	0.101
	RL (Mean)	0.044	0.054
	RL (Mean + 1σ)	0.109	0.127
	RL (Mean + 2σ)	0.162	0.243
CH_4_ adsorption at 0.9 bar (mol/kg)	Original Data	0.818	0.623
	RL (Mean)	1.224	0.745
	RL (Mean + 1σ)	1.342	0.760
	RL (Mean + 2σ)	1.834	1.004
CO_2_ adsorption at 0.1 bar (mol/kg)	Original Data	0.599	0.682
	RL (Mean)	0.576	0.657
	RL (Mean + 1σ)	0.637	0.563
	RL (Mean + 2σ)	0.432	0.522
Band gap (eV)	Original Data	1.944	1.027
	RL (Mean – 1σ)	1.523	0.769
	RL (Mean)	1.289	0.577
	RL (Mean + 1σ)	1.767	0.520
	RL (Mean + 2σ)	2.044	0.83

a Fine-tuned model achieved 0% validity; so the associated statistics are not shown in this table.

To demonstrate the broad applicability of our framework, we conducted additional validation experiments across the complete range of pressure conditions in our data set. While our primary results focus on select conditions, we also validated our approach across all available pressure conditions (detailed results provided in Supporting Information). This cross-pressure consistency demonstrates that our RL framework captures fundamental structure–property relationships rather than overfitting to specific experimental conditions.

To validate the robustness of our multicomponent reward design, we conducted a systematic sensitivity analysis examining how ± 33% perturbations in key reward weights affect optimization performance. Using CH_4_ adsorption at 0.05 bar as a representative case, we evaluated target achievement across three difficulty levels (mean, mean+1σ, mean+2σ) under varied reward configurations. The analysis demonstrates that our baseline reward weights provide optimal performance stability, with both weight increases and reductions leading to performance degradation (shown in Supporting Information). This validates that our chosen parameter configuration represents a robust optimum that generalizes across diverse targeting scenarios without requiring task-specific retuning.

#### Sample Analysis of Generated MOF Structures

To further our understanding of the type of outputs generated by our RL framework, we conduct a sample analysis of generated structures across various tasks. This analysis reveals consistent trends in how our RL framework explores chemical space across different operational regimes. Across property-specific targets, the model learns to assemble metal nodes and organic linkers into topologies consistent with known high-performing motifs, while also generating novel compositions.

Preliminary analysis of the generated building blocks shows they consist of well-known, synthetically accessible organic linkers (carboxylates, nitrogen-containing heterocycles) paired with common metal nodes (Zn^2+^, Cu^2+^ paddle wheels), suggesting reasonable synthetic feasibility for experimental validation. The generated MOFs across tasks, a sample of which are shown in Table 3 feature diverse topologies (e.g., pcu, nbo) and exhibit both common and unconventional linker-metal combinations. For instance, while Zn-based and Cu-based clusters are frequently selected, their pairing with alkynes, halogens, or amine-functionalized linkers shows some novelty beyond the training distribution.

**Table 3:** Representative Examples of RL-Generated MOF Structures with Target Properties

**Target**	**Generated MOFid**	**Predicted Property**
High CH_4_ adsorption at 0.5 bar (mol/kg)	*CCCOc*1*cc*(*cc*(*c*1*C*(= *O*)​[*O*−])​*OCCC*)​*c*1*ccc*(*cc*1)​*c*1*cc*(*OCCC*)​*c*(*c*(*c*1)*OCCC*)​*C*(= *O*)​[*O*−].[*O*−]​*C*(= *O*)​*c*1*ccc*(*cc*1)​*C*(= *O*)​[*O*−].[*Zn*]​[*Zn*]*&* *&nbo*.*cat*0	2.414 mol/kg
High CO_2_ adsorption at 0.5 bar (mol/kg)	*CCCC*(= *CC*(= *O*)​[*O*−])​*C* = *CC*(= *O*)​[*O*−].*CCCC*1*CC*2​(*CCC*1​(*CC*2)​*C*(= *O*)​[*O*−])​*C*(= *O*)​[*O*−].*CCC*[*N*]​[*CH*]​[*CH*]​[*NH*].[*Cu*]​[*Cu*]*&* *&pcu*.*cat*0	3.33 mol/kg
Low Band Gap (eV)	*N#CC#N*.​[*O*−]​*C*(= *O*)​*C#CC#CC*(= *O*)​[*O*−].​[*O*−]​*C*(= *O*)​*C*12*C*3*C*4​(*C*2(*C*2*C*1​(*C*3(*C*42*C*(= *O*)[*O*−])​*Cl*)*Cl*)*Cl*)​*Cl*.[*Zn*][*Zn*]​*&* *&pcu*.*cat*1	0.435 eV

Our analysis reveals that the RL framework successfully identifies structure–property correlations that drive enhanced performance. For high CH_4_ uptake targets, generated MOFs show preference for rigid aromatic linkers bearing carboxylates, combined with high-porosity, low-catenation topologies such as nbo, and Zn^2+^ nodes known for forming stable, spacious cages. This is consistent with prior studies showing enhanced uptake in high-porosity, carboxylate-rich frameworks.^10,72^ This suggests the RL objective is capturing relevant structure–property correlations rather than producing random outliers.

High CO_2_ uptake structures consistently feature open Cu^2+^ paddle wheel units and nitrogen- or oxygen-rich linkers, enabling stronger electrostatic and Lewis acid–base interactions.^73,74^ In contrast, low band gap optimization yields MOFs with strong π-conjugation, donor–acceptor functionality (e.g., – *C#N*, – *C#C* triple bonds, Cl halogens), and π-stacking potential enabled by interpenetrated pcu.cat1 networks, together creating low-energy frontier orbitals and reduced band gaps.^75–77^

While these high-performing structures represent promising candidates for experimental realization, comprehensive stability assessment through DFT calculations and synthetic accessibility evaluation would be valuable next steps. The MOFid representation enables reconstruction of 3D crystal structures through current MOFid-to-CIF conversion protocols,^28^ allowing for subsequent geometric optimization and detailed characterization of the most promising candidates.

## Conclusions

In this work, we introduced a reinforcement learning-enhanced GPT-based generative framework for target-specific de novo MOF design. Our approach integrates a GPT-based MOF generator with property-aware feedback through a transformer-based property predictor, using a comprehensive reward function that balances validity, novelty, diversity, and property targeting. The framework addresses the challenge of generating chemically valid MOFs while controlling their functional properties.

A key finding of this work is identifying an important limitation in current generative design approaches that our RL framework helps to address. While fine-tuned models consistently generate invalid structures across tasks, our RL-optimized approach guides MOF generation toward specific property targets while maintaining reasonable validity rates, with structural diversity exceeding 83%. This difference between fine-tuned and RL approaches represents a meaningful improvement in computational materials design methodology.

Building on this foundation, the observed shifts in property distributions toward challenging targets demonstrate the effectiveness of our approach in navigating the complex chemical space of MOFs. The targeting performance analysis shows controlled optimization over MOF property landscapes across three distinct domains. Our framework demonstrates good targeting performance for moderate objectives, achieving improvements in CH_4_ adsorption targets and systematic band gap reduction for enhanced conductivity applications. Even for extreme targets corresponding to the top 4.5% of property performance in the original data set, the framework maintains appreciable validity rates while generating more than 70% novel structures.

The ability to target properties in the tail of the distribution (mean+2σ) while maintaining reasonable validity suggests that our approach can explore underrepresented regions of chemical space, potentially leading to the discovery of MOFs with enhanced properties that would be difficult to identify through traditional screening approaches. Importantly, this targeting capability extends across different property domains without requiring domain-specific modifications. Cross-domain validation across gas adsorption and electronic properties establishes the framework’s applicability and demonstrates that the underlying RL methodology can optimize diverse functional characteristics using the same computational infrastructure with minimal changes. This versatility is particularly valuable for materials applications requiring multiproperty optimization.

While these results are promising, some limitations must be acknowledged. The generated MOFid strings represent chemically reasonable combinations of building blocks and topologies but do not guarantee that the resulting structures are thermodynamically stable or experimentally realizable. Future work could incorporate stability assessments through density functional theory (DFT) calculations or machine learning-based stability predictors to filter generated candidates. Additionally, synthetic accessibility could be evaluated by comparing generated building blocks against experimentally reported MOF components or by implementing retrosynthetic analysis tools adapted for MOF chemistry. Integration with 3D structure construction and physics-based validation tools would further enhance the practical utility of our approach.

Additionally, while we demonstrate clear improvements over our supervised fine-tuning baseline, comprehensive benchmarking against other MOF-specific generative models from the literature would strengthen our evaluation. Such comparisons are complicated by differences in representation schemes, training data sets, and evaluation protocols across different studies. Future work should focus on establishing standardized benchmarking protocols for MOF generative models to enable fair comparisons across different approaches.

Beyond these immediate improvements, MOFGPT represents a modular and reusable RL framework that decouples generation from property prediction, enabling flexible substitution of value heads or reward criteria. While demonstrated on MOFid strings, the same paradigm may extend to other structured materials, such as COFs or zeolites. This work contributes to a shift from screening existing materials to designing new ones with predetermined characteristics. The reinforcement learning framework developed here provides a foundation that could be extended to other materials design tasks, with future work potentially focusing on integrating feedback from physics-based simulations and multimodal approaches to further enhance the design of high-performance MOFs.

## Supplementary Material



## Data Availability

The necessary information containing the code and data used in this study is available here: https://github.com/srivathsanb14/MOFGPT.

## Notes

Detailed MOFid tokenization procedures, comprehensive data set information, complete model architecture and three-stage training methodology, multicomponent reward function design, generation and evaluation protocols, and additional property optimization results across all 11 conditions including CH_4_ and CO_2_ adsorption at multiple pressures and electronic band gap targeting. (PDF)
