# Supplementary material for: MOFGPT: Generative Design of Metal–Organic Frameworks using Language Models
Source: J Chem Inf Model. 2025 Aug 28;65(17):9049–60. doi: 10.1021/acs.jcim.5c01625 (PMC12421666; doi:10.1021/acs.jcim.5c01625)
Supplement: Supplementary file 1 [file ci5c01625_si_001.pdf]

Supporting Information: MOFGPT: Generative Design of Metal-Organic Frameworks using Language Models

Srivathsan Badrinarayanan,[†] Rishikesh Magar,[‡] Akshay Antony,[‡] Radheesh Sharma Meda,[†] and Amir Barati Farimani[*,‡,¶,†,§]

†*Department of Chemical Engineering, Carnegie Mellon University, 15213, USA*

‡*Department of Mechanical Engineering, Carnegie Mellon University, 15213, USA*

¶*Department of Biomedical Engineering, Carnegie Mellon University, 15213, USA*

§*Machine Learning Department, Carnegie Mellon University, 15213, USA*

E-mail: barati@cmu.edu

Data Representation and Preprocessing

MOFid Representation and Tokenization

Our work utilizes a specialized dual-component tokenizer designed to interpret Metal-Organic Framework identifiers (MOFids), which encapsulates both chemical and structural information in a concise format. This representation follows the approach introduced by Bucior et al.,[S1] where MOFid consists of two primary components: the SMILES notation[S2] of secondary building units (SBUs) and the topology codes from the Reticular Chemistry Structure Resource (RCSR) database.[S3]

The tokenization process employs a two-stage pipeline to account for the distinct characteristics of chemical and topological information. For the SMILES component, we imple-

mented a regex-based tokenizer derived from the molecular transformer work of Schwaller et al.,[S4,S5] which effectively captures atomic symbols, bonds, branches, and other chemical notation elements. This approach ensures precise tokenization of complex chemical structures within MOFs. The topology component undergoes separate processing through a specialized tokenizer that parses comma-separated topology codes and optional categorical information denoted after period delimiters.

Our vocabulary is derived from a comprehensive source originally developed for the USPTO dataset,[S6] which provides extensive coverage of chemical tokens while maintaining a manageable vocabulary size for efficient model training. The tokenizer manages special tokens including beginning-of-sequence, end-of-sequence, padding, masking, and unknown tokens to facilitate transformer-based sequence processing. Chemical and topological information are clearly delineated using a separator token ”&&” between the components, preserving the logical structure of the MOFid representation. Table S1 presents representative examples of MOFid representations for various types of MOFs, illustrating how the format captures both chemical composition through SMILES strings and topological information. Following the BERT approach,[S7] our tokenizer adds a [CLS] token and a [SEP] token (equivalent to [BOS] and [EOS] in our implementation) at the beginning and end of the sequence, respectively, to symbolize the start and end of the MOF representation.

Table S1: Examples of MOFid Representations for Various Metal-Organic Frameworks

**Example 1:** Copper node with benzenedicarboxylate linker in primitive cubic topology<br>C1=CC(=CC=C1C(=O)O)C(=O)O.Cu && pcu
**Example 2:** Copper-based MOF with mixed linkers (nitro-functionalized and unfunctionalized benzoates)<br>c1cc(cc(c1)C(=O)O)N(=O)=O.c1cc(cc(c1)C(=O)O)C(=O)O.Cu && nbo-d
**Example 3:** Zinc-based MOF with benzonitrile linkers in diamondoid topology with catenation<br>C1=CC=C(C=C1)C#N.Zn && dia-c

All MOF representations are standardized to a maximum sequence length of 512 tokens

through padding or truncation as needed. Our analysis confirmed that this approach effectively preserves structural information, with only 0.37% of structures in the hMOF dataset exceeding the token limit. For these rare cases, the truncation preserves the most chemically relevant information while maintaining computational efficiency. During model training, appropriate attention masking techniques ensure that padding tokens do not contribute to the learned representations.

The tokenization pipeline supports batch processing with dynamic padding based on the longest sequence in each batch, optimizing computational efficiency during training. This approach transforms the MOF structural and chemical information into a format suitable for autoregressive sequence modeling while maintaining the critical relationships between building units and topological arrangements that define MOF functionality.

Datasets and Property Representations

Dataset Composition and Training Split

Our study aggregates MOF structures from three comprehensive, publicly available repositories for GPT pretraining purpose: Boyd & Woo,[S8] quantum MOF (QMOF),[S9,S10] and hypothetical MOF (hMOF)[S11] datasets. Following deduplication and shuffling procedures, we created a training set of 323,469 samples ($\sim$ 80%) and a held-out test set of 81,260 samples ($\sim$ 20%). Table S2 provides the detailed composition of our dataset split across the three sources. The Boyd & Woo dataset constitutes the majority of our corpus, while the hMOF dataset provides about a quarter of the samples, and the QMOF dataset contributes a smaller but significant portion. This diverse combination ensures our model learns from a broad spectrum of MOF architectures and chemical compositions.

Property Data for Reinforcement Learning

Our reinforcement learning framework leverages two complementary datasets with distinct property domains to enable comprehensive MOF design capabilities: the hMOF dataset for

Table S2: Composition of the pretraining (train) and test datasets by source.

**Dataset**	**Train**	**Test**	%Train	%Test
Boyd & Woo	235,841	58,950	73.0%	72.5%
hMOF	82,114	20,744	25.4%	25.5%
QMOF	5,514	1,566	1.7%	1.9%
**Total**	323,469	81,260	100%	100%

gas adsorption properties and the QMOF dataset for electronic properties.

The hMOF dataset contains gas adsorption property measurements across various conditions. Our dataset systematically captures both the target gas (CO_2 or CH_4) and the pressure conditions (ranging from 0.01 to 4.5 bar) at which adsorption properties were simulated. A key strength of this dataset lies in the fact that all adsorption properties were calculated using the same set of MOF structures but under different simulation conditions. This approach yielded 10 distinct property datasets (5 pressure points for each of the two gases) while maintaining structural consistency across all 82,114 training and 20,744 test MOF structures.

The pressure points were strategically selected to span the entire application spectrum for MOF-based gas systems: ultra-low pressures (0.01-0.05 bar) relevant for trace gas detection and sensing applications; medium pressures (0.1-0.9 bar) applicable to post-combustion carbon capture and environmental remediation; and higher pressures (2.5-4.5 bar) pertinent to gas storage and separation technologies. All adsorption values are reported in $mol.kg^{-1}$, with higher values indicating superior adsorption performance which is a critical consideration for applications such as carbon capture and gas storage where maximizing capacity is often a primary objective.

On the other hand, the QMOF dataset provides DFT-calculated band gap values measured in electron volts (eV) for each MOF structure.[S12,S13] Band gap is a critical electronic property in materials science, with lower values indicating better conductivity, which is a characteristic particularly valuable for energy storage applications and electronic devices.

This dataset offers an opportunity to evaluate and optimize for electronic properties that involve complex quantum mechanical phenomena, broadening the application scope of our reinforcement learning framework beyond traditional gas adsorption targets.

Our comprehensive evaluation encompasses multiple property prediction tasks across different pressure conditions. Specifically, we utilize 5 CH_4 adsorption datasets at pressures of 0.05, 0.5, 0.9, 2.5, and 4.5 bar, 5 CO_2 adsorption datasets at pressures of 0.01, 0.05, 0.1, 0.5, and 2.5 bar, and 1 electronic band gap dataset from QMOF. For our primary results demonstration in the main text, we selected representative conditions that span low-pressure (0.05 bar CH_4, 0.01 bar CO_2), high-pressure (0.9 bar CH_4), and electronic properties (band gap) to showcase the framework's versatility across different physical regimes and property types. The selection of these specific conditions was strategically made to represent practically relevant pressure regimes: 0.05 bar for CH_4 represents low-pressure storage applications relevant to natural gas vehicles, 0.9 bar captures near-atmospheric conditions important for industrial separation processes, and 0.01 bar for CO_2 represents trace CO_2 capture scenarios critical for atmospheric remediation applications. Complete results for all 11 property prediction tasks are presented in Appendix A, demonstrating consistent performance trends with validity rates maintained above 35% and novelty rates exceeding 60% across all conditions.

Together, these datasets provide diverse objective functions for our reinforcement learning framework, enabling the generation of MOF structures with enhanced performance across multiple application domains. For all benchmark datasets used in targeted property generation, we followed established protocols by splitting them into training, validation, and test sets with a ratio of 0.8:0.05:0.15.

Model Architecture and Training Methodology

Base Language Model

Our MOFGPT framework utilizes a transformer-based architecture derived from the GPT-2 model, adapted specifically for processing MOFid representations. The model consists of 12 transformer decoder layers with an embedding dimension of 768, 12 attention heads, and a feed-forward dimension of 3072. The architecture follows the standard transformer decoder design with self-attention mechanisms, feed-forward neural networks, and residual connections. The transformer decoder employs a self-attention mechanism defined by:

$$\text{Attention}(Q, K, V) = \text{softmax}\left(\frac{QK^T}{\sqrt{d_k}} + M\right) V \tag{1}$$

where Q, K, and V are the query, key, and value matrices derived from the input embeddings, d_k is the dimension of the key vectors, and M is the attention mask that prevents attending to future tokens during training (causal attention). Each transformer layer incorporates a multi-head attention mechanism and position-wise feed-forward network with residual connections and layer normalization. The complete model configuration parameters are detailed in Table S3.

Table S3: Base Model Configuration Parameters

**Parameter**	**Value**
Number of layers	12
Hidden dimension	768
Feed-forward dimension	3072
Number of attention heads	12
Maximum sequence length	512
Vocabulary size	4023
Dropout rate	0.1
Activation function	SiLU (Swish)
Layer normalization epsilon	1e-5

Model Training Pipeline

Our implementation follows a three-stage training approach:

Pretraining

We first pretrain the base language model using a next-token prediction objective on a large corpus of MOF structures. The pretraining objective is to maximize the log-likelihood of the next token given the previous tokens:

$$\mathcal{L}_{\text{pretrain}} = -\sum_{t=1}^{T} \log P_\theta(x_t | x_{<t}) \tag{2}$$

where x_t is the token at position t, $x_{<t}$ represents all tokens before position t, and P_θ is the probability distribution over the vocabulary given by the model with parameters θ.

Fine-tuning

After pretraining, we fine-tune the model for property prediction using supervised learning. We extend the base language model with a specialized regression head that processes the output embeddings from the transformer decoder through a series of feed-forward layers with nonlinear activations to extract property-relevant features. The fine-tuning objective is to minimize the mean squared error between the predicted and actual property values:

$$\mathcal{L}_{\text{finetune}} = \frac{1}{N} \sum_{i=1}^{N} (y_i - \hat{y}_i)^2 \tag{3}$$

where y_i is the ground truth property value for the i-th MOF, $\hat{y}_i$ is the predicted value, and N is the number of training examples. During fine-tuning, we employ several techniques to improve model performance, including learning rate scheduling with warm-up, gradient clipping, and early stopping based on validation loss.

Reinforcement Learning

The reinforcement learning phase optimizes the model to generate MOFs with specific target properties. We employ the REINFORCE algorithm (policy gradient method) with several enhancements to improve training stability and efficiency. The RL model consists of two main components: (1) Policy network, which is the pretrained language model that defines the probability distribution over possible outputs at each step (2) Value network, which is the fine-tuned property prediction model that estimates the expected reward for a given MOF sequence. The RL objective is to maximize the expected reward:

$$J(\theta) = \mathbb{E}_{\tau \sim \pi_\theta}[R(\tau)] \tag{4}$$

where π_θ is the policy defined by the model parameters θ and $R(\tau)$ is our multi-component reward function. The gradient of this objective can be estimated using the policy gradient theorem:

$$\nabla_\theta J(\theta) \approx \frac{1}{B} \sum_{i=1}^{B} \sum_{t=1}^{T_i} \nabla_\theta \log \pi_\theta(a_t^i | s_t^i) \cdot R(\tau^i) \cdot \gamma^{T_i - t} \tag{5}$$

where B is the batch size, T_i is the length of the i-th sequence, a_t^i is the token selected at step t for sequence i, s_t^i is the corresponding state, $R(\tau^i)$ is the reward for the complete sequence, and γ is a discount factor that gives greater weight to rewards associated with earlier actions.

We implement several enhancements to the basic REINFORCE algorithm. Our training employs a global memory system that tracks the highest-performing MOF structures across all epochs, enabling the model to maintain awareness of promising chemical space regions without requiring explicit experience replay mechanisms. We add a KL divergence term to prevent the policy from deviating too far from the pretrained model, ensuring that generated structures remain chemically sensible:

$$\mathcal{L}_{\text{prox}} = \beta \cdot D_{\text{KL}}(\pi_\theta || \pi_{\theta_0}) \tag{6}$$

where β is a weighting factor and π_{θ_0} is the initial policy from the pretrained model. We subtract a baseline from the rewards to reduce variance in gradient estimates:

$$\nabla_\theta J(\theta) \approx \frac{1}{B} \sum_{i=1}^{B} \sum_{t=1}^{T_i} \nabla_\theta \log \pi_\theta(a_t^i | s_t^i) \cdot (R(\tau^i) - b) \tag{7}$$

where b is a baseline value, often chosen as the mean reward of the batch. We employ temperature scheduling and top-k/top-p sampling to balance exploration and exploitation during generation. We gradually increase the influence of property-specific rewards as training progresses to prevent premature convergence to suboptimal solutions. The complete RL training algorithm is provided in Algorithm 1. The hyperparameters for each training stage are detailed in Table S4. As shown in Table S4, we used a smaller learning rate for the RL phase (1e-5) compared to pretraining (1e-3) to ensure stable policy updates while maintaining the model's pretrained knowledge.

Table S4: Detailed Model Hyperparameters for Different Training Phases

**Hyperparameter**	**Pretraining**	**Fine-tuning**	**RL Training**
Learning rate	1e-3	1e-4	1e-5
Batch size	128	8	32
Warmup ratio	0.03	0.03	0.03
Scheduler	Cosine	None	Cosine
Weight decay	0.001	0.001	0.0001
Gradient accumulation steps	1	1	1
Maximum sequence length	512	512	512
Dropout rate	0.1	0.1	0.1
Epochs	30	30	60
**RL-Specific Parameters**			
Discount factor (γ)	-	-	0.99
Novelty factor (α_{nov})	-	-	1.5
Validity factor (α_{val})	-	-	2.5
Diversity factor (α_{div})	-	-	2.0
Target weights (α_{tgt})	-	-	3.0
Evaluation interval	-	-	5 epochs

Algorithm 1 Reinforcement Learning Training Procedure

Input: Pretrained model π_{θ_0}, property predictor f, target properties p^*
Output: Optimized model π_θ
Initialize model parameters: $\theta \leftarrow \theta_0$
Initialize global memory: $\mathcal{M}_{\text{global}} \leftarrow \emptyset$
for epoch $e = 1$ to E **do**
  Generate batch of sequences $\{\tau_1, \tau_2, ..., \tau_B\} \sim \pi_\theta$
  Convert sequences to MOF representations $\{m_1, m_2, ..., m_B\}$
  Predict properties $\{\hat{p}_1, \hat{p}_2, ..., \hat{p}_B\}$ using predictor f
  Calculate rewards $\{R(\tau_1), R(\tau_2), ..., R(\tau_B)\}$ using multi-component reward function
  Update global memory $\mathcal{M}_{\text{global}}$ with top-performing structures
  Select top-K sequences based on target rewards for focused learning
  Compute policy gradients using REINFORCE algorithm
  Update model parameters θ using computed gradients
  if evaluation interval reached **then**
    Evaluate model on validation set
    Save checkpoint if performance improved
  end if
end for
return Optimized model π_θ

Reward Function Design

This section provides comprehensive details of our multi-component reward function that guides MOF generation toward structures with desired properties while maintaining chemical validity, novelty, and structural diversity. Our implementation incorporates global memory management and adaptive mechanisms to address key challenges in molecular generation such as mode collapse and maintaining exploration throughout training.

Design Philosophy and Challenges

The design of our multi-component reward function addresses fundamental challenges unique to MOF generative modeling that distinguish it from small molecule generation. While small organic molecules can usually be validated through established chemical rules, MOFs require additional consideration of both local coordination chemistry and global topological consistency. This challenge necessitated our comprehensive reward architecture that balances

multiple competing objectives while maintaining focus on property targeting.

Traditional reward functions in molecular generation often suffer from several critical issues. First, focusing solely on target properties can cause mode collapse, where the model generates only a few high-reward structures repeatedly. Second, balancing exploration (novelty and diversity) with exploitation (target optimization) requires careful design. Third, maintaining learning signals for all generated structures while focusing on the most promising candidates is essential for stable training.

Our reward function addresses these challenges through three key innovations: (1) global memory that preserves high-performing structures across epochs, (2) multi-component diversity rewards that prevent mode collapse while maintaining focus on target properties, and (3) adaptive top-K selection that focuses learning on promising structures while maintaining gradient flow.

Reward Architecture and Implementation

Our reward function integrates four fundamental components, each addressing a specific aspect of successful molecular generation. The implementation uses a straightforward architecture that balances target property achievement with exploration and validity constraints. The total reward calculation follows a simple additive approach with multiplicative bonuses:

For structures selected in the top-K performers:

$$R_{\text{total}}(m) = R_{\text{target}}(m) \cdot \beta_{\text{base}} \cdot M_{\text{valid}}(m) \cdot M_{\text{novel}}(m) + R_{\text{novelty}}(m) + R_{\text{validity}}(m) + R_{\text{diversity}}(m) \tag{8}$$

For structures not in the top-K:

$$R_{\text{total}}(m) = R_{\text{target}}(m) \cdot \alpha_{\text{reduced}} \tag{9}$$

where $\beta_{\text{base}} = 3.0$ is a fixed base multiplier, $\alpha_{\text{reduced}} = 0.3$ is the reduced reward factor,

and the multiplicative components are applied individually:

$$M_{\text{valid}}(m) = \begin{cases} 1.1 & \text{if structure is valid} \\ 1.0 & \text{otherwise} \end{cases} \tag{10}$$

$$M_{\text{novel}}(m) = \begin{cases} 1.1 & \text{if structure is novel} \\ 1.0 & \text{otherwise} \end{cases} \tag{11}$$

The additive bonus components provide small independent rewards for exploration:

$$R_{\text{novelty}}(m) = \text{novelty\_factor} \times \nVdash_{\text{novel}}(m) \times 0.1 \tag{12}$$

$$R_{\text{validity}}(m) = \text{validity\_factor} \times \nVdash_{\text{valid}}(m) \times 0.1 \tag{13}$$

$$R_{\text{diversity}}(m) = \text{diversity\_factor} \times S_{\text{diversity}}(m) \times 0.1 \tag{14}$$

The top-K selection mechanism focuses learning on the most promising structures while maintaining gradient flow for all generated molecules. The selection ratio decreases from 50% early in training to 30% in later stages to become increasingly selective as the model improves.

Core Reward Components

Target Property Reward

The target property reward is the core component that drives optimization toward desired property values. This component receives the highest weighting because property optimization is the primary objective of the generation process:

$$R_{\text{target}}(m) = \sum_{i=1}^{k} w_i \cdot R_{\text{proximity}}(\hat{p}_i(m), T_i) \tag{15}$$

where $\hat{p}_i(m)$ is the predicted value of property i for structure m, T_i is the target value

for property i, and w_i is the importance weight for property i.

The proximity reward calculation provides rewards based on relative distance to target:

$$R_{\text{proximity}}(\hat{p}, T) = R_{\text{base}}(\delta_{\text{rel}}) \cdot f_{\text{direction}}(\hat{p}, T) \cdot w \tag{16}$$

where $\delta_{\text{rel}} = \frac{|\hat{p}-T|}{|T|+\epsilon}$ is the relative distance to target, with $\epsilon = 1 \times 10^{-6}$ for numerical stability. The base reward function provides a tiered reward structure that matches the implementation:

$$R_{\text{base}}(\delta_{\text{rel}}) = \begin{cases} 15.0 & \text{if } \delta_{\text{rel}} \leq 0.05 \\ 12.0 & \text{if } 0.05 < \delta_{\text{rel}} \leq 0.1 \\ 8.0 & \text{if } 0.1 < \delta_{\text{rel}} \leq 0.2 \\ 4.0 & \text{if } 0.2 < \delta_{\text{rel}} \leq 0.5 \\ \max(1.0, 4.0(1-\delta_{\text{rel}})) & \text{if } \delta_{\text{rel}} > 0.5 \end{cases} \tag{17}$$

The direction factor provides incentives for optimization in the desired direction:

$$f_{\text{direction}}(\hat{p}, T) = \begin{cases} 1.3 & \text{if optimization achieved in correct direction} \\ 1.1 & \text{if close to target (within 20\% buffer)} \\ 0.95 & \text{otherwise} \end{cases} \tag{18}$$

For "higher" optimization mode, the achievement condition is $\hat{p} \geq T$, while for "lower" mode it is $\hat{p} \leq T$. The close-to-target condition applies when the prediction is within 80-120% of the target value depending on the optimization direction.

Validity Reward

The validity reward ensures that generated structures adhere to chemical and structural constraints:

$$R_{\text{validity}}(m) = \nVdash_{\text{valid}}(m) = \begin{cases} 1 & \text{if valid according to validation procedure} \\ 0 & \text{otherwise} \end{cases} \tag{19}$$

Our validation procedure consists of multiple checks designed specifically for MOF structures: SMILES syntax validation using RDKit with metal atom substitution for compatibility, metal node presence verification, structural balance confirmation requiring both organic and inorganic components, topology validity against known RCSR database entries when topology tokens are present, and coordination number validation for metal centers. The validity component appears both as a small additive bonus (scaled by 0.1) and as a multiplicative enhancement (factor of 1.1) when applied to top-K structures.

Novelty Reward

The novelty reward encourages exploration of new regions of chemical space rather than reproducing training data structures:

$$R_{\text{novelty}}(m) = \nVdash_{\text{novel}}(m) = \begin{cases} 1 & \text{if } m \notin \mathcal{D}_{\text{train}} \\ 0 & \text{otherwise} \end{cases} \tag{20}$$

where $\mathcal{D}_{\text{train}}$ represents the set of MOF structures in the training dataset. Novelty checking involves comparing the generated MOFid representation with all training examples using exact string matching. Like validity, novelty also appears as both a small additive bonus (scaled by 0.1) and a multiplicative enhancement (factor of 1.1) for top-K structures.

Diversity Reward

The diversity reward prevents mode collapse by encouraging variety in generated structures. This component incorporates multiple diversity metrics to capture different aspects of structural variety:

$$R_{\text{diversity}}(m, \mathcal{B}, \mathcal{H}) = w_b \cdot S_{\text{batch}}(m, \mathcal{B}) + w_n \cdot S_{\text{ngram}}(m, \mathcal{B}) + w_h \cdot S_{\text{history}}(m, \mathcal{H}) + w_c \cdot S_{\text{composition}}(m) \tag{21}$$

where $\mathcal{B}$ is the current batch, $\mathcal{H}$ is the generation history, and the weights are $w_b = 0.30$, $w_n = 0.25$, $w_h = 0.35$, and $w_c = 0.10$.

Batch Diversity Component: Measures structural differences between MOFs in the same generation batch using approximate distance metrics:

$$S_{\text{batch}}(m, \mathcal{B}) = \frac{2}{|\mathcal{B}| - 1} \sum_{m' \in \mathcal{B}, m' \neq m} d_{\text{approx}}(m, m') \tag{22}$$

where $d_{\text{approx}}(m, m')$ combines string length differences and character-wise differences for computational efficiency.

N-gram Diversity Component: Analyzes character n-grams (length 4) to identify overused structural patterns:

$$S_{\text{ngram}}(m, \mathcal{B}) = \min\left(1.0, \frac{1.0}{\text{AvgFreq}(m, \mathcal{B})/|\mathcal{B}| + \epsilon}\right) \tag{23}$$

Historical Uniqueness Component: Prevents cycling by maintaining memory of previously generated structures:

$$S_{\text{history}}(m, \mathcal{H}) = \begin{cases} 0.1 & \text{if } m \in \mathcal{H} \text{ (exact duplicate)} \\ \frac{1}{\max(1, C_{\text{sig}}(m, \mathcal{H}))} & \text{otherwise} \end{cases} \tag{24}$$

where $C_{\text{sig}}(m, \mathcal{H})$ counts structures with similar signatures in the generation history.

Compositional Diversity Component: Encourages variety in chemical building blocks:

$$S_{\text{composition}}(m) = \min\left(1.0, 0.5 \cdot \frac{|\text{Elements}(m)|}{10} + 0.5 \cdot \frac{|\text{FunctionalGroups}(m)|}{5}\right) \tag{25}$$

Advanced Reward Mechanisms

Global Memory and Cross-Epoch Learning

To prevent loss of high-performing structures and enable learning from past discoveries, we maintain a global memory of the best structures across all training epochs:

$$\mathcal{M}_{\text{global}} = \{(m_j, p_j, s_j, r_j)\}_{j=1}^{N_{\text{mem}}} \tag{26}$$

where each entry contains a MOF structure m_j, its predicted properties p_j, target progress score s_j, and total reward r_j, with $N_{\text{mem}} = 200$. The target score provides a unified ranking metric that considers progress toward all objectives:

$$s_j = \sum_{i=1}^{k} w_i \cdot \text{Progress}_i(p_{j,i}, T_i) \tag{27}$$

The progress function rewards both achievement and over-achievement in the desired direction:

$$\text{Progress}_i(p, T) = \begin{cases} 1.0 + \frac{p-T}{|T|} & \text{if mode=''higher'' and } p \geq T \\ \max(0.1, \frac{p}{T}) & \text{if mode=''higher'' and } p < T \\ 1.0 + \frac{T-p}{|T|} & \text{if mode=''lower'' and } p \leq T \\ \max(0.1, \frac{T}{p}) & \text{if mode=''lower'' and } p > T \end{cases} \tag{28}$$

This formulation ensures that structures exceeding targets receive bonuses proportional to their over-achievement, while structures making progress toward targets receive credit proportional to their advancement.

Training Optimization Mechanisms

Top-K Selection and Reward Focusing

To focus learning on promising structures, we implement an adaptive top-K selection mechanism:

$$N_{\text{top-k}}(e) = \max\left(3, \lfloor N_{\text{batch}} \cdot r_{\text{top-k}}(e) \rfloor\right) \tag{29}$$

The top-K ratio becomes more selective as training progresses:

$$r_{\text{top-k}}(e) = \begin{cases} 0.5 & \text{if } e < 100 \\ 0.4 & \text{if } 100 \leq e < 200 \\ 0.3 & \text{if } e \geq 200 \end{cases} \tag{30}$$

For structures not selected in the top-K, we provide a reduced reward signal:

$$R_{\text{reduced}}(m) = \max(0.1, 0.3 \cdot R_{\text{target}}(m)) \tag{31}$$

Reward Normalization and Stability

To prevent gradient explosion while preserving signal relationships, we apply conditional normalization only when rewards exceed reasonable bounds:

$$R_{\text{normalized}}(r) = \begin{cases} \mu_{\text{target}} + \frac{r-\mu_R}{\sigma_R} \cdot \sigma_{\text{target}} & \text{if } \mu_R > 100 \text{ or } \sigma_R > 50 \\ r & \text{otherwise} \end{cases} \tag{32}$$

where $\mu_{\text{target}} = 20.0$ and $\sigma_{\text{target}} = 10.0$ are conservative normalization parameters.

A minimum reward guarantee ensures gradient flow:

$$R_{\text{final}}(m) = \max(0.1, R_{\text{normalized}}(m)) \tag{33}$$

Implementation Parameters

Table S5: Implemented Reward Function Parameters

Parameter	Value	Description
**Core Component Weights**		
Base target multiplier (β_{base})	3.0	Fixed multiplier for target rewards
Individual bonus scaling factors	0.1	Applied separately to novelty, validity, diversity
Reduced reward factor (α_{reduced})	0.3	For non-top-K structures
**Validity/Novelty Multipliers**		
Valid structure multiplier	1.1	Applied when structure is valid
Novel structure multiplier	1.1	Applied when structure is novel
**Target Proximity Rewards**		
Excellent ($\delta_{\text{rel}} \leq 0.05$)	15.0	Highest base reward tier
Very good ($0.05 < \delta_{\text{rel}} \leq 0.1$)	12.0	Second reward tier
Good ($0.1 < \delta_{\text{rel}} \leq 0.2$)	8.0	Third reward tier
Moderate ($0.2 < \delta_{\text{rel}} \leq 0.5$)	4.0	Fourth reward tier
Achievement direction bonus	1.3	Target achieved in correct direction
Close to target bonus	1.1	Within 20% of target
Wrong direction penalty	0.95	Mild penalty for opposite direction
**Diversity Component Weights**		
Batch diversity (w_b)	0.30	Immediate batch-level variety
N-gram diversity (w_n)	0.25	Structural pattern diversity
Historical diversity (w_h)	0.35	Prevention of long-term cycling
Compositional diversity (w_c)	0.10	Chemical building block variety
Exact duplicate penalty	0.1	Heavy penalty for exact matches
**Global Memory and Selection**		
Global memory size (N_{mem})	200	Maximum stored structures
Early top-K ratio (epochs < 100)	0.5	Top 50% selection
Mid top-K ratio (100-200)	0.4	Top 40% selection
Late top-K ratio (≥ 200)	0.3	Top 30% selection
Minimum top-K count	3	Ensures gradient flow
**Stability and Normalization**		
Generation history size	500	For diversity calculation
N-gram size	4	Character subsequence length
Normalization mean threshold	100.0	Triggers normalization
Normalization std threshold	50.0	Triggers normalization
Target normalization mean	20.0	Conservative rescaling target
Target normalization std	10.0	Conservative rescaling spread
Minimum reward guarantee	0.1	Prevents zero rewards

The parameter values as shown in Table S5 represent a balance between target achievement, chemical validity, structural diversity, and training stability, enabling successful op-

timization across MOF design challenges while maintaining computational efficiency and training stability.

Generation and Evaluation

Model Inference Pipelines

Our framework provides two distinct inference pathways tailored to the specific characteristics of fine-tuned and reinforcement learning-optimized models. Each pipeline incorporates specialized generation strategies, validation protocols, and evaluation metrics designed to maximize the effectiveness of the respective model types.

Fine-tuned Model Inference

The fine-tuned model inference pipeline focuses on generating diverse MOF structures while maintaining chemical validity and providing accurate property predictions. The generation process begins with the [BOS] token and employs autoregressive sampling to produce complete MOF sequences:

$$\text{Sequence} = \{[BOS], t_1, t_2, \ldots, t_T, [EOS]\} \tag{34}$$

where each token t_i is sampled from the probability distribution:

$$P(t_i|t_{<i}) = \text{softmax}\left(\frac{f_\theta(t_{<i})}{\tau}\right) \tag{35}$$

where f_θ represents the fine-tuned model's output logits, τ is the temperature parameter controlling sampling diversity, and $t_{<i}$ denotes all previous tokens in the sequence.

The fine-tuned inference pipeline implements several key components:

Sequence Generation: We employ nucleus sampling (top-p) combined with temperature scaling to balance exploration and coherence. The generation continues until either the

[EOS] token is produced or the maximum sequence length (512 tokens) is reached. Multiple sequences are generated in parallel to improve efficiency and provide diverse candidates.

Property Prediction: For each generated MOF sequence, we leverage the same fine-tuned model to predict target properties. The sequence is re-tokenized and passed through the model's regression head to obtain property estimates:

$$\hat{p} = f_{\text{pred}}(\text{Embed}(\text{MOF}_{\text{tokens}})) \tag{36}$$

where f_{pred} is the property prediction head and Embed represents the token embedding and transformer encoding process.

Validation and Filtering: Generated MOFs undergo multi-stage validation including SMILES syntax verification using RDKit (with metal substitution for compatibility), structural completeness checks ensuring both organic and inorganic components are present, and novelty assessment against the training dataset to identify genuinely new structures.

Reinforcement Learning Model Inference

The RL model inference pipeline is specifically designed to generate MOFs that achieve target property values while maintaining diversity and chemical validity. This pipeline incorporates the sophisticated reward mechanisms developed during training to guide generation toward high-performing structures.

Policy-Guided Generation: The RL model operates as a learned policy π_θ that has been optimized to maximize expected rewards for target properties. During inference, we sample from this policy using controlled stochasticity:

$$a_t \sim \pi_\theta(\cdot|s_t) \tag{37}$$

where a_t is the selected token at step t and s_t is the current sequence state.

Multi-Component Evaluation: Each generated MOF is evaluated using the same

multi-component reward function employed during training, providing comprehensive assessment across validity, novelty, diversity, and target property achievement. This evaluation helps rank generated structures and identify the most promising candidates.

Curriculum-Informed Sampling: The RL inference process can optionally incorporate examples from the global memory accumulated during training, using high-performing historical structures to guide generation toward promising regions of chemical space.

Advanced Filtering Mechanisms: Our RL inference pipeline includes optional relaxed filtering for structures with high predicted property values. This mechanism applies more lenient validity and novelty criteria to promising MOFs that might otherwise be discarded due to minor structural irregularities:

$$\text{Accept}(\text{MOF}) = \begin{cases} \text{Standard\_Filter}(\text{MOF}) & \text{if } \hat{p} < \theta \cdot p_{\text{target}} \\ \text{Relaxed\_Filter}(\text{MOF}) & \text{if } \hat{p} \geq \theta \cdot p_{\text{target}} \end{cases} \tag{38}$$

where θ is a threshold factor (typically 0.8) and p_{target} is the target property value.

Generation Process Parameters

The generation process employs carefully tuned parameters to optimize the balance between exploration and exploitation. Table S6 provides comprehensive parameter settings for both model types.

Table S6: Detailed Generation Parameters for Model Inference

**Parameter**	**Fine-tuned Model**	**RL-optimized Model**
Temperature	0.7	0.7
Top-k sampling	400	100
Top-p (nucleus) sampling	0.9	0.9
Beam size	1	1
Early stopping	True	True
Max sequence length	512	512
Batch size	20	50
Number of return sequences	Variable	32

Temperature Control: Both models use a temperature of 0.7, providing a balance between deterministic generation (which might lead to repetitive structures) and highly stochastic sampling (which could produce invalid sequences). This temperature was selected through empirical evaluation to maximize both diversity and validity.

Sampling Strategy Differences: The fine-tuned model uses more permissive top-k sampling (400 tokens) to encourage exploration of diverse chemical space, while the RL model uses more restrictive top-k sampling (100 tokens) to focus on high-reward regions identified during training.

Batch Processing: Generation is performed in batches to improve computational efficiency while maintaining memory constraints. The RL model uses larger batch sizes to better leverage the reward-based ranking mechanisms.

Evaluation Metrics

Our evaluation framework employs multiple complementary metrics to assess generation quality across different dimensions of MOF design success.

Structural Validity Rate: The percentage of generated MOFs that pass comprehensive chemical validation:

$$R_{\text{validity}} = \frac{\sum_{i=1}^{N} \nVdash_{\text{valid}}(m_i)}{N} \times 100\% \tag{39}$$

where $\nVdash_{\text{valid}}(m_i)$ indicates whether MOF m_i passes all validation checks including SMILES syntax correctness, presence of both organic and inorganic components, metal coordination feasibility, and topology consistency (when applicable). We verify that generated MOFs contain appropriate ratios of organic linkers and metal nodes, ensuring chemical plausibility of the proposed structures.

Novelty Rate: The fraction of valid MOFs that are absent from the training dataset:

$$R_{\text{novelty}} = \frac{\sum_{i=1}^{N_{\text{valid}}} \mathbb{1}_{\text{novel}}(m_i)}{N_{\text{valid}}} \times 100\% \tag{40}$$

Structural Diversity: We quantify diversity through multiple complementary measures including unique structure count, average pairwise distance within generated sets, and n-gram pattern analysis to detect repetitive motifs:

$$D_{\text{diversity}} = \frac{|\text{Unique}(\mathcal{M}_{\text{generated}})|}{|\mathcal{M}_{\text{generated}}|} \tag{41}$$

Target Proximity: For structures with predicted properties, we measure proximity to target values:

$$S_{\text{proximity}} = \frac{1}{N} \sum_{i=1}^{N} \exp\left(-\frac{|\hat{p}_i - p_{\text{target}}|}{p_{\text{target}}}\right) \tag{42}$$

Property Distribution Analysis: Comprehensive statistical analysis of generated property distributions including mean, median, standard deviation, and comparison with training data distributions.

Generation Efficiency Metrics

Overall Efficiency: The fraction of generation attempts that result in valid, novel MOFs meeting target criteria:

$$E_{\text{overall}} = \frac{N_{\text{valid}} \cap N_{\text{novel}} \cap N_{\text{target}}}{N_{\text{attempted}}} \times 100\% \tag{43}$$

Computational Efficiency: We track generation time per MOF, memory usage, and convergence characteristics to assess practical deployment feasibility.

Inference-Time Enhancements

During inference, we implement an optional relaxed filtering mechanism that applies more lenient validity and novelty criteria to MOF candidates with high predicted property values. This inference-time enhancement helps recover potentially valuable structures that might be discarded due to minor technical violations while maintaining strict standards for typical candidates.

The relaxed filtering is applied when:

$$\hat{p}_i \geq \theta \cdot p_{\text{target}} \quad \text{where } \theta = 0.8 \tag{44}$$

For such high-property candidates, the system applies:

- **Relaxed Validity**: Uses RDKit parsing without full sanitization, allowing recovery of chemically reasonable but technically non-standard structures
- **Relaxed Novelty**: Permits structures with high similarity (>85%) to training data if they represent meaningful chemical variations

This approach balances the need for chemical validity with the exploration of high-performance chemical space regions that might be missed by overly strict filtering criteria.

Limitations, Future Work, and Applications

Limitations

While our approach demonstrates strong performance in generating MOFs with targeted properties, several limitations should be noted. The reinforcement learning optimization is only as good as the underlying property prediction model; inaccuracies in property prediction can lead to suboptimal targeting.[S14,S15] Our approach generates MOFs in the form of MOFid

representations, which capture chemical composition and topology but not detailed 3D coordinates; a separate step is required to generate the full 3D structure, which may introduce additional uncertainty.[S1,S16] While our model generates chemically valid structures, it does not explicitly consider synthetic accessibility or stability under realistic conditions.[S17,S18] The model's generations are influenced by the distribution of structures in the training data, which may limit exploration of novel regions of chemical space.[S19] The reinforcement learning training process is computationally intensive, requiring significant GPU resources and time.[S20]

Future Work

Based on the limitations identified, several directions for future work emerge. Integrating more sophisticated property prediction models, such as graph neural networks or equivariant neural networks, could improve targeting accuracy.[S21,S22] Extending the framework to directly generate 3D structures would eliminate the need for a separate structure generation step and potentially improve property prediction accuracy.[S23] Adding synthetic accessibility and stability criteria to the reward function could lead to more practically useful MOF candidates.[S16,S17] Implementing an active learning loop that iteratively refines the property prediction model based on feedback from high-fidelity simulations or experiments would improve accuracy over time.[S21] Exploring more sample-efficient reinforcement learning algorithms, such as proximal policy optimization (PPO) or soft actor-critic (SAC), could reduce computational requirements.[S24,S25] Extending the framework to simultaneously optimize multiple properties with potentially conflicting objectives would address more complex design challenges.[S26,S27]

Potential Applications

The MOFGPT framework enables the targeted design of MOFs for a wide range of applications. For gas storage and separation, it allows designing MOFs with optimized capacity and

selectivity for CH_4, CO_2, H_2, and other gases, with applications in natural gas storage, carbon capture, and hydrogen economy.[S28–S30] In catalysis, the framework can generate MOFs with specific band gaps and active sites for heterogeneous catalysis, photocatalysis, and electrocatalysis.[S31,S32] For sensing applications, it can create MOFs with tailored guest-host interactions for chemical sensing and detection of specific analytes.[S33] In drug delivery, the system can develop biocompatible MOFs with controlled pore sizes and functionalities for medical applications.[S34] For electronic and optical materials, our approach can design MOFs with specific electronic and optical properties for applications in optoelectronics, semiconductors, and energy conversion.[S35]

Appendix: Additional Property Optimization Results

This appendix presents comprehensive results for reinforcement learning-based MOF generation across eight additional property domains: four CH_4 adsorption conditions at different pressures and four CO_2 adsorption conditions at different pressures. The results follow the same evaluation framework as the main text, demonstrating the robustness and versatility of our approach across diverse operating conditions. It should be noteworthy again that the finetuned distributions correspond to completely invalid generations.

Methane Adsorption Optimization at Multiple Pressures

We evaluated our RL approach for CH_4 adsorption optimization at five pressure conditions: 0.05, 0.5, 0.9, 2.5, and 4.5 bar.

CH_4 Adsorption at 0.05 bar

Table S7: Performance Metrics for RL-Based MOF Generation - CH_4 Adsorption at 0.05 bar

Target	Validity (%)	Novelty (%)	Diversity (%)
Mean	49.2	90.9	97
Mean + 1σ	39.5	83.33	97
Mean + 2σ	58.3	85.71	97

Table S8: Statistical Properties of Generated Structures - CH_4 Adsorption at 0.05 bar

Dataset	Mean	Std Dev
Original Data	0.066	0.101
Fine-tuned	1.131	0.730
RL (Mean)	0.044	0.054
RL (Mean + 1σ)	0.109	0.127
RL (Mean + 2σ)	0.162	0.243

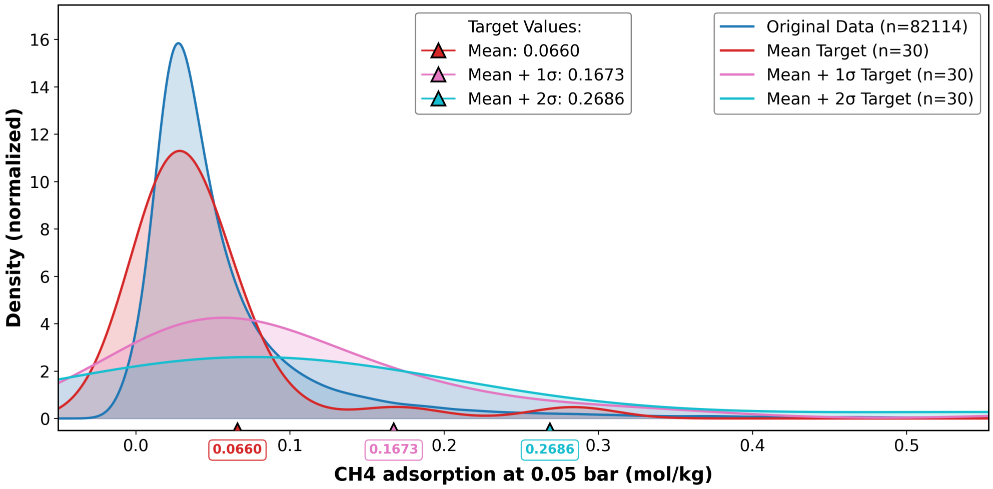

Figure S1: Normalized density distributions of CH_4 adsorption at 0.05 bar showing systematic targeting across mean, mean + 1σ, and mean + 2σ scenarios.

CH_4 Adsorption at 0.5 bar

Table S9: Performance Metrics for RL-Based MOF Generation - CH_4 Adsorption at 0.5 bar

Target	Validity (%)	Novelty (%)	Diversity (%)
Mean	89.13	73.17	100
Mean + 1σ	55.2	71.4	89.0
Mean + 2σ	20.35	88.32	99.5

Table S10: Statistical Properties of Generated Structures - CH_4 Adsorption at 0.5 bar

Dataset	Mean	Std Dev
Original Data	0.507	0.449
Fine-tuned	0.177	0.439
RL (Mean)	0.824	0.570
RL (Mean + 1σ)	0.732	0.581
RL (Mean + 2σ)	0.441	0.256

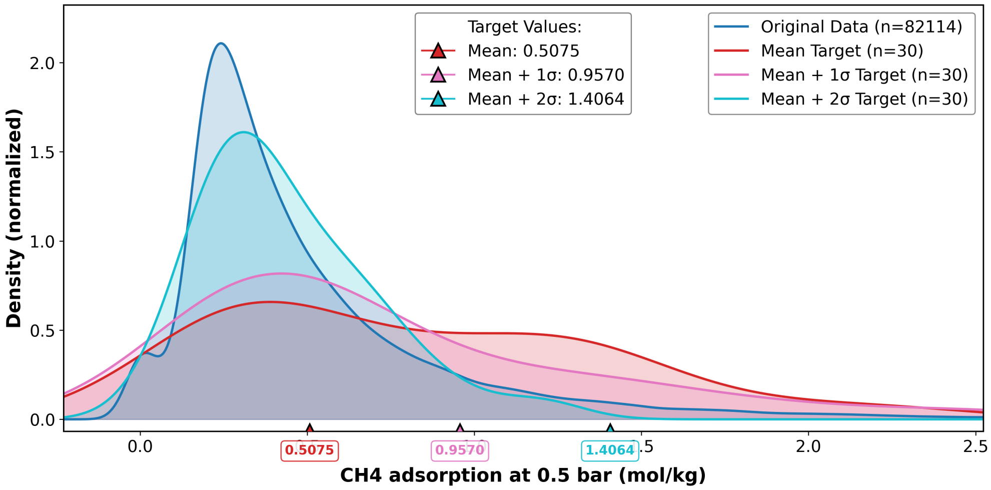

Figure S2: Normalized density distributions of CH_4 adsorption at 0.5 bar.

CH_4 Adsorption at 0.9 bar

Table S11: Performance Metrics for RL-Based MOF Generation - CH_4 Adsorption at 0.9 bar

Target	Validity (%)	Novelty (%)	Diversity (%)
Mean	71.21	63.82	100
Mean + 1σ	52	76.92	99
Mean + 2σ	53.42	76.92	97

Table S12: Statistical Properties of Generated Structures - CH_4 Adsorption at 0.9 bar

Dataset	Mean	Std Dev
Original Data	0.818	0.623
Fine-tuned	0.608	0.769
RL (Mean)	1.224	0.745
RL (Mean + 1σ)	1.342	0.760
RL (Mean + 2σ)	1.834	1.004

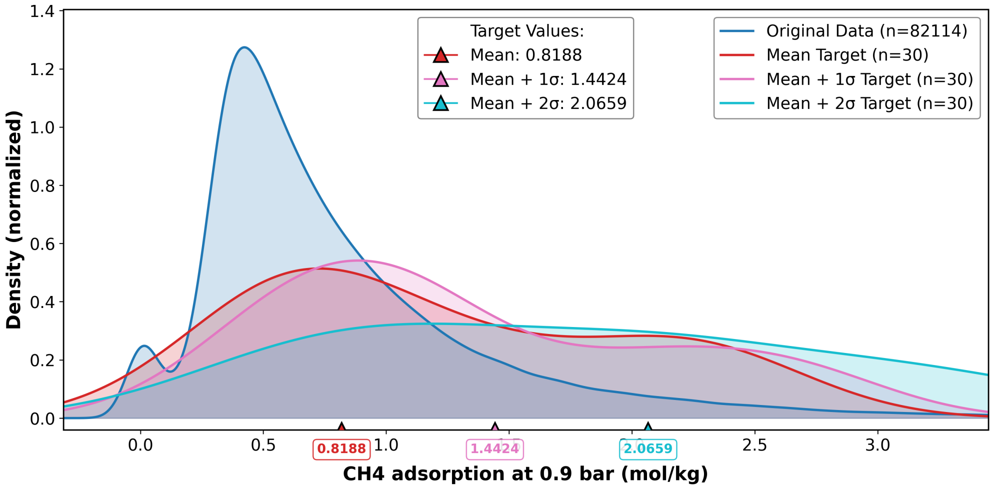

Figure S3: Normalized density distributions of CH_4 adsorption at 0.9 bar.

CH_4 Adsorption at 2.5 bar

Table S13: Performance Metrics for RL-Based MOF Generation - CH_4 Adsorption at 2.5 bar

Target	Validity (%)	Novelty (%)	Diversity (%)
Mean	82.35	53.57	100
Mean + 1σ	56	71.42	100
Mean + 2σ	64.28	86.11	99

Table S14: Statistical Properties of Generated Structures - CH_4 Adsorption at 2.5 bar

Dataset	Mean	Std Dev
Original Data	1.788	1.044
Fine-tuned	1.421	1.391
RL (Mean)	2.447	1.089
RL (Mean + 1σ)	2.149	1.142
RL (Mean + 2σ)	2.568	1.152

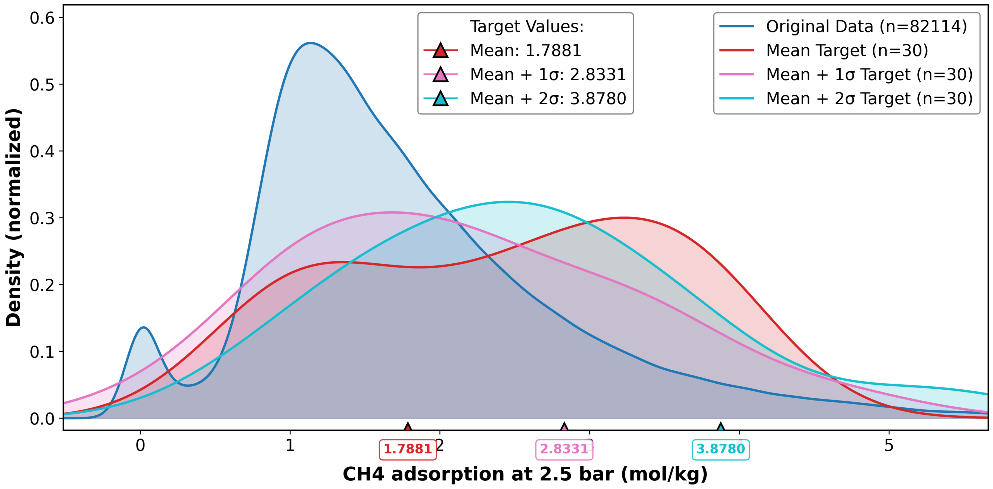

Figure S4: Normalized density distributions of CH_4 adsorption at 2.5 bar.

CH_4 Adsorption at 4.5 bar

Table S15: Performance Metrics for RL-Based MOF Generation - CH_4 Adsorption at 4.5 bar

Target	Validity (%)	Novelty (%)	Diversity (%)
Mean	23.94	58.82	100
Mean + 1σ	62.33	62.5	99
Mean + 2σ	60.52	65.21	98

Table S16: Statistical Properties of Generated Structures - CH_4 Adsorption at 4.5 bar

Dataset	Mean	Std Dev
Original Data	2.708	1.377
Fine-tuned	0.530	0.882
RL (Mean)	2.485	1.236
RL (Mean + 1σ)	3.836	1.620
RL (Mean + 2σ)	3.863	1.225

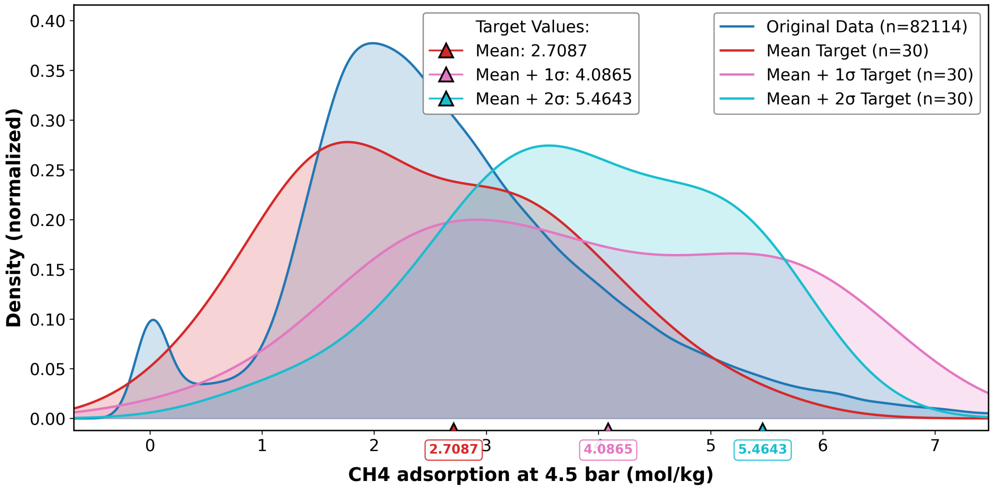

Figure S5: Normalized density distributions of CH_4 adsorption at 4.5 bar.

Carbon Dioxide Adsorption Optimization at Multiple Pressures

Similarly, we evaluated CO_2 adsorption optimization at five pressure conditions: 0.01, 0.05, 0.1, 0.5 and 2.5 bar.

CO_2 Adsorption at 0.01 bar

Table S17: Performance Metrics for RL-Based MOF Generation - CO_2 Adsorption at 0.01 bar

Target	Validity (%)	Novelty (%)	Diversity (%)
Mean	54.23	93.75	100
Mean + 1σ	44.87	88.57	99
Mean + 2σ	58.82	100	99

Table S18: Statistical Properties of Generated Structures - CO_2 Adsorption at 0.01 bar

Dataset	Mean	Std Dev
Original Data	0.102	0.218
Fine-tuned	2.849	0.521
RL (Mean)	0.228	0.466
RL (Mean + 1σ)	0.052	0.048
RL (Mean + 2σ)	0.142	0.174

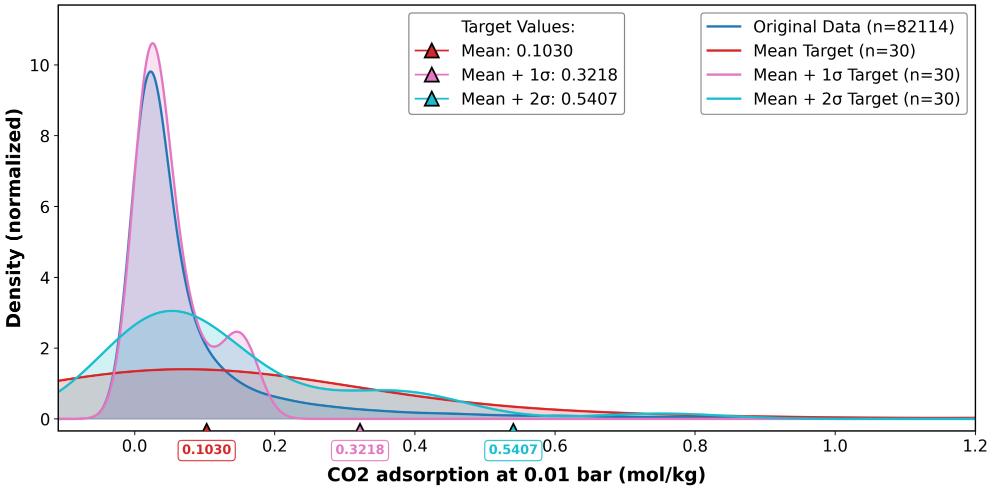

Figure S6: Normalized density distributions of CO_2 adsorption at 0.01 bar.

CO_2 Adsorption at 0.05 bar

Table S19: Performance Metrics for RL-Based MOF Generation - CO_2 Adsorption at 0.05 bar

Target	Validity (%)	Novelty (%)	Diversity (%)
Mean	90	83.33	100
Mean + 1σ	43.90	88.88	86
Mean + 2σ	70.21	90.90	100

Table S20: Statistical Properties of Generated Structures - CO_2 Adsorption at 0.05 bar

Dataset	Mean	Std Dev
Original Data	0.358	0.488
Fine-tuned	1.023	2.452
RL (Mean)	0.334	0.397
RL (Mean + 1σ)	0.814	0.765
RL (Mean + 2σ)	0.468	0.345

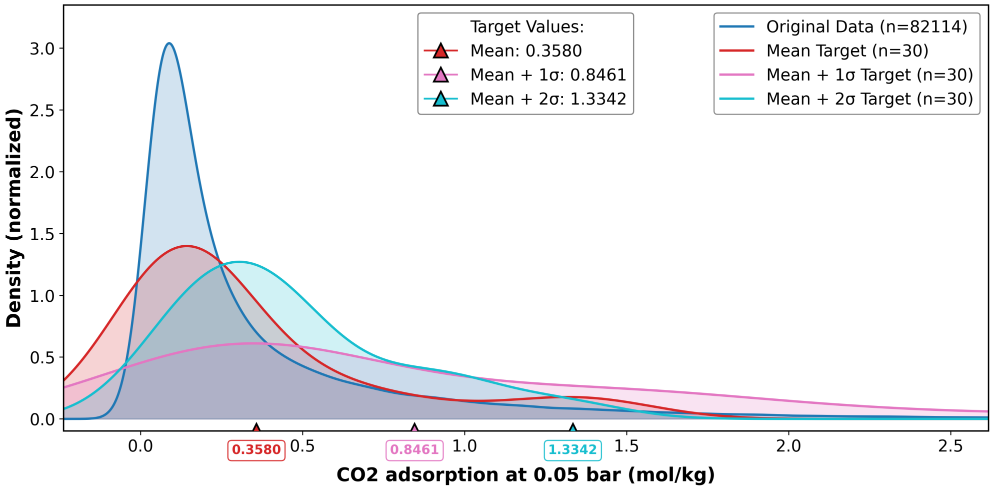

Figure S7: Normalized density distributions of CO_2 adsorption at 0.05 bar.

CO_2 Adsorption at 0.1 bar

Table S21: Performance Metrics for RL-Based MOF Generation - CO_2 Adsorption at 0.1 bar

Target	Validity (%)	Novelty (%)	Diversity (%)
Mean	42.85	83.33	99
Mean + 1σ	50	83.33	99
Mean + 2σ	100	100	100

Table S22: Statistical Properties of Generated Structures - CO_2 Adsorption at 0.1 bar

Dataset	Mean	Std Dev
Original Data	0.599	0.682
Fine-tuned	-0.177	0.746
RL (Mean)	0.576	0.657
RL (Mean + 1σ)	0.637	0.563
RL (Mean + 2σ)	0.432	0.522

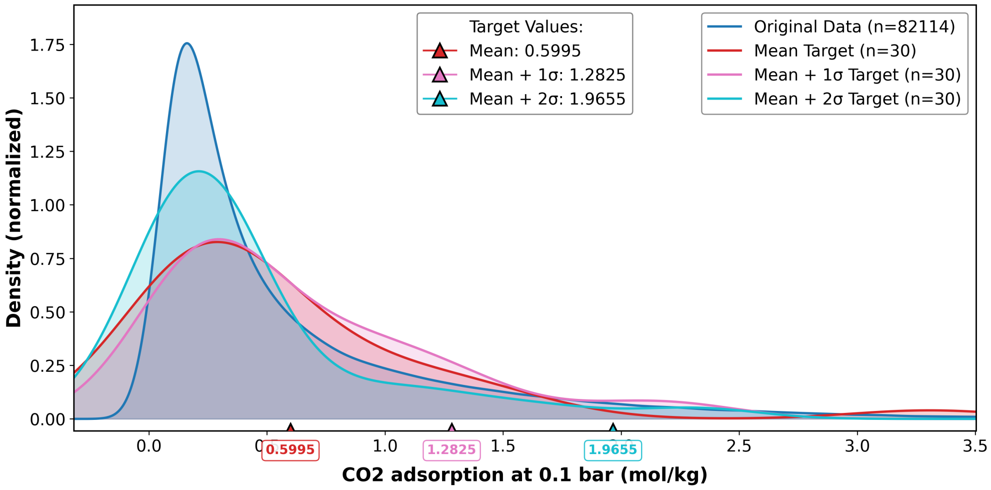

Figure S8: Normalized density distributions of CO_2 adsorption at 0.1 bar.

CO_2 Adsorption at 0.5 bar

Table S23: Performance Metrics for RL-Based MOF Generation - CO_2 Adsorption at 0.5 bar

Target	Validity (%)	Novelty (%)	Diversity (%)
Mean	95.12	76.9	100
Mean + 1σ	65	76.92	100
Mean + 2σ	15.81	81.08	100

Table S24: Statistical Properties of Generated Structures - CO_2 Adsorption at 0.5 bar

Dataset	Mean	Std Dev
Original Data	1.849	1.403
Fine-tuned	1.943	2.268
RL (Mean)	1.324	0.680
RL (Mean + 1σ)	2.383	1.117
RL (Mean + 2σ)	1.696	1.006

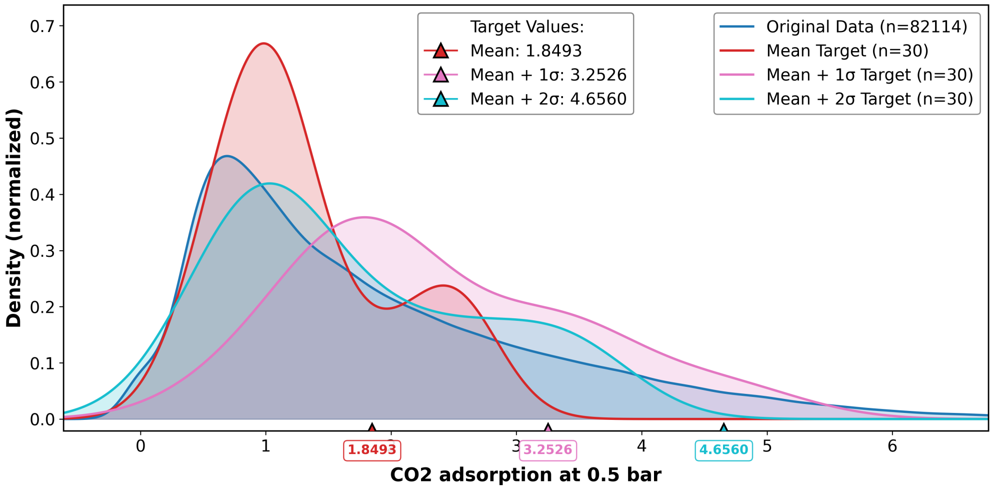

Figure S9: Normalized density distributions of CO_2 adsorption at 0.5 bar.

CO_2 Adsorption at 2.5 bar

Table S25: Performance Metrics for RL-Based MOF Generation - CO_2 Adsorption at 2.5 bar

Target	Validity (%)	Novelty (%)	Diversity (%)
Mean	57.42	51.72	99.33
Mean + 1σ	66.55	62.5	99
Mean + 2σ	63.63	85.71	99

Table S26: Statistical Properties of Generated Structures - CO_2 Adsorption at 2.5 bar

Dataset	Mean	Std Dev
Original Data	5.138	2.706
Fine-tuned	4.421	1.885
RL (Mean)	5.944	3.032
RL (Mean + 1σ)	6.320	2.535
RL (Mean + 2σ)	5.463	2.006

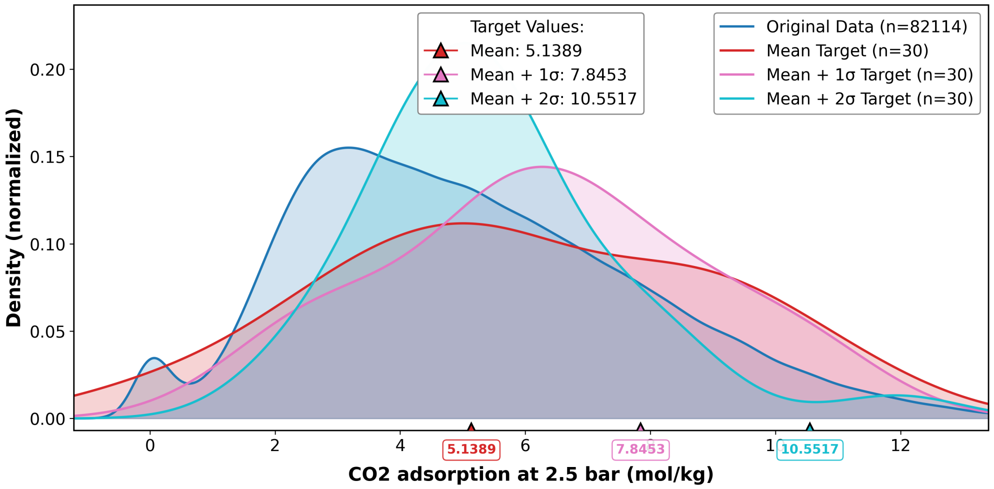

Figure S10: Normalized density distributions of CO_2 adsorption at 2.5 bar.

Band gap (eV)

We also evaluated band gap energy optimization (eV).

Table S27: Performance Metrics for RL-Based MOF Generation - Band gap (eV)

Target	Validity (%)	Novelty (%)	Diversity (%)
Mean	100	93.75	93.75
Mean + 1σ	64	93.75	100
Mean + 2σ	35.63	100	83.0
Mean - 1σ	39.75	90.90	100

Table S28: Statistical Properties of Generated Structures - Band gap (eV)

Dataset	Mean	Std Dev
Original Data	1.944	1.027
Fine-tuned	1.961	0.984
RL (Mean - 1σ)	1.523	0.769
RL (Mean)	1.289	0.577
RL (Mean + 1σ)	1.767	0.520
RL (Mean + 2σ)	2.044	0.83

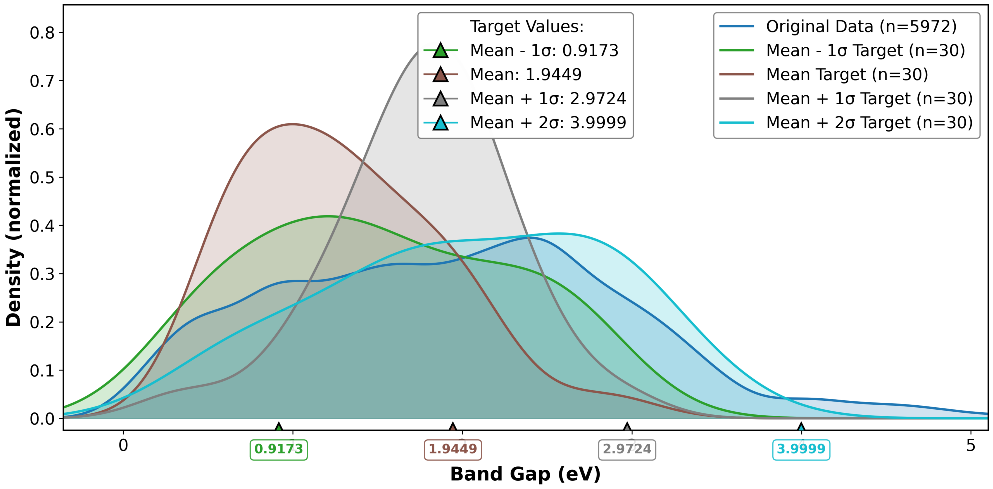

Figure S11: Normalized density distributions of Band gap (eV).

Summary of Multi-Pressure Performance

Table S29: Summary of Average Performance Metrics Across All Pressure Conditions

Gas	Pressure Range	Avg Validity (%)	Avg Novelty (%)	Avg Diversity (%)
CH_4	0.05-4.5 bar	55.8 ± 17.9	73.9 ± 11.1	98.1 ± 2.7
CO_2	0.01-2.5 bar	61.2 ± 21.2	83.1 ± 12.4	98.6 ± 3.4

The comprehensive evaluation across multiple pressure conditions demonstrates the robustness of our reinforcement learning approach for MOF property optimization. Consistent performance across different operating pressures validates the framework's applicability to diverse applications.

Reward Function Sensitivity Analysis

To validate the robustness of our multi-component reward function design, we conducted a sensitivity analysis examining how perturbations in key reward weights affect the optimization performance. We systematically varied the target reward weights by $\sim \pm 30\%$ from their baseline values and evaluated the resulting impact on generated MOF property distributions using the CH_4 adsorption at 0.05 bar dataset.

The baseline reward configuration used target weights of 9.0, which we perturbed to 12.0 (+33%) and 6.0 (-33%). For each perturbation scenario, we generated MOF structures targeting three different property values: mean (0.066 mol·kg^{-1}), mean + 1σ (0.167 mol·kg^{-1}), and mean + 2σ (0.268 mol·kg^{-1}). The analysis focused on how these weight variations affect the model's ability to achieve the specified target values.

The sensitivity analysis reveals several key findings about the reward function stability. For the mean target scenario, increasing the reward weights by 33% resulted in generated values (0.068 mol·kg^{-1}) that closely approximated the target (0.066 mol·kg^{-1}), representing only a 3% deviation. In contrast, decreasing the weights by 33% led to a significant deviation (0.268 mol·kg^{-1}), indicating that insufficient reward weighting can compromise target

Table S30: Sensitivity Analysis Results for Target Weight Perturbations

Target Scenario	Weight Change	Target Value	Generated Mean
Mean	+33%	0.066	0.068
	Baseline (0%)	0.066	0.044
	-33%	0.066	0.268
Mean + 1σ	+33%	0.167	0.097
	Baseline (0%)	0.167	0.109
	-33%	0.167	0.035
Mean + 2σ	+33%	0.268	0.058
	Baseline (0%)	0.268	0.162
	-33%	0.268	0.059

achievement. For higher target scenarios (mean + 1σ and mean + 2σ), the weight perturbations produced more complex responses. The -33% weight reduction specifically led to substantial underperformance relative to the desired targets.

These results demonstrate that while our reward function exhibits directional sensitivity to parameter changes, it maintains reasonable performance bounds across moderate perturbations. Notably, for extreme target values (mean + 2σ), the perturbed weights show significant performance degradation: both +33% and -33% perturbations resulted in generated means (0.058 and 0.059 mol·kg^{-1}, respectively) substantially below the target (0.268 mol·kg^{-1}), while the baseline configuration achieved much better approximation (0.162 mol·kg^{-1}). This breakdown at extreme values demonstrates that our chosen baseline weights represent an optimal balance for robust performance across diverse targeting scenarios.

The framework shows greatest stability when reward weights are maintained at or mildly above baseline values, suggesting that our chosen parameter selection provides robust optimization performance. It is important to note that our framework is designed to provide generalizable weight configurations that perform reasonably across diverse MOF optimization tasks. While users can further tune these parameters to their specific datasets and targeting requirements, our baseline configuration offers a robust starting point that maintains per-

formance stability across a wide range of property values and optimization scenarios. This analysis validates the appropriateness of our chosen reward function weights and provides guidance for parameter selection in related optimization scenarios.

References

(S1) Bucior, B. J.; Rosen, A. S.; Haranczyk, M.; Yao, Z.; Ziebel, M. E.; Farha, O. K.; Hupp, J. T.; Siepmann, J. I.; Aspuru-Guzik, A.; Snurr, R. Q. Identification Schemes for Metal–Organic Frameworks To Enable Rapid Search and Cheminformatics Analysis. *Crystal Growth & Design* **2019**, *19*, 6682–6697.

(S2) Weininger, D. SMILES, a chemical language and information system. 1. Introduction to methodology and encoding rules. *Journal of Chemical Information and Computer Sciences* **1988**, *28*, 31–36.

(S3) O'Keeffe, M.; Peskov, M. A.; Ramsden, S. J.; Yaghi, O. M. The Reticular Chemistry Structure Resource (RCSR) Database of, and Symbols for, Crystal Nets. *Accounts of Chemical Research* **2008**, *41*, 1782–1789, PMID: 18834152.

(S4) Schwaller, P.; Laino, T.; Gaudin, T.; Bolgar, P.; Hunter, C. A.; Bekas, C.; Lee, A. A. Molecular Transformer: A Model for Uncertainty-Calibrated Chemical Reaction Prediction. *ACS Central Science* **2019**, *5*, 1572–1583, PMID: 31572784.

(S5) Schwaller, P.; Gaudin, T.; Lányi, D.; Bekas, C.; Laino, T. "Found in Translation": predicting outcomes of complex organic chemistry reactions using neural sequence-to-sequence models. *Chem. Sci.* **2018**, *9*, 6091–6098.

(S6) Schwaller, P.; Probst, D.; Vaucher, A. C.; Nair, V. H.; Kreutter, D.; Laino, T.; Reymond, J.-L. Mapping the space of chemical reactions using attention-based neural networks. *Nature Machine Intelligence* **2021**, *3*, 144–152.

(S7) Devlin, J.; Chang, M.-W.; Lee, K.; Toutanova, K. BERT: Pre-training of Deep Bidirectional Transformers for Language Understanding. 2019; https://arxiv.org/abs/1810.04805.

(S8) Boyd, P. G.; Chidambaram, A.; García-Díez, E.; Ireland, C. P.; Daff, T. D.; Bounds, R.; Gładysiak, A.; Schouwink, P.; Moosavi, S. M.; Maroto-Valer, M. M.; others Data-driven design of metal–organic frameworks for wet flue gas CO2 capture. *Nature* **2019**, *576*, 253–256.

(S9) Rosen, A. S.; Iyer, S. M.; Ray, D.; Yao, Z.; Aspuru-Guzik, A.; Gagliardi, L.; Notestein, J. M.; Snurr, R. Q. Machine learning the quantum-chemical properties of metal–organic frameworks for accelerated materials discovery. *Matter* **2021**, *4*, 1578–1597.

(S10) Rosen, A. S.; Fung, V.; Huck, P.; O'Donnell, C. T.; Horton, M. K.; Truhlar, D. G.; Persson, K. A.; Notestein, J. M.; Snurr, R. Q. High-throughput predictions of metal–organic framework electronic properties: theoretical challenges, graph neural networks, and data exploration. *npj Computational Materials* **2022**, *8*, 1–10.

(S11) Wilmer, C. E.; Leaf, M.; Lee, C. Y.; Farha, O. K.; Hauser, B. G.; Hupp, J. T.; Snurr, R. Q. Large-scale screening of hypothetical metal–organic frameworks. *Nature chemistry* **2012**, *4*, 83–89.

(S12) Xie, L. S.; Skorupskii, G.; Dincă, M. Electrically Conductive Metal–Organic Frameworks. *Chemical Reviews* **2020**, *120*, 8536–8580, PMID: 32275412.

(S13) Sheberla, D.; Bachman, J. C.; Elias, J. S.; Sun, C.-J.; Shao-Horn, Y.; Dincă, M. Conductive MOF electrodes for stable supercapacitors with high areal capacitance. *Nature materials* **2017**, *16*, 220–224.

(S14) Wang, Z.; Zhou, Y.; Zhou, T.; Sundmacher, K. Identification of optimal metal-organic

frameworks by machine learning: Structure decomposition, feature integration, and predictive modeling. *Computers & Chemical Engineering* **2022**, *160*, 107739.

(S15) Altintas, C.; Altundal, O. F.; Keskin, S.; Yildirim, R. Machine Learning Meets with Metal Organic Frameworks for Gas Storage and Separation. *Journal of Chemical Information and Modeling* **2021**, *61*, 2131–2146, PMID: 33914526.

(S16) Cao, Z.; Magar, R.; Wang, Y.; Barati Farimani, A. MOFormer: Self-Supervised Transformer Model for Metal–Organic Framework Property Prediction. *Journal of the American Chemical Society* **2023**, *145*, 2958–2967, PMID: 36706365.

(S17) Nandy, A.; Yue, S.; Oh, C.; Duan, C.; Terrones, G. G.; Chung, Y. G.; Kulik, H. J. A database of ultrastable MOFs reassembled from stable fragments with machine learning models. *Matter* **2023**, *6*, 1585–1603.

(S18) Butova, V. V.; Soldatov, M. A.; Guda, A. A.; Lomachenko, K. A.; Lamberti, C. Metal-organic frameworks: structure, properties, methods of synthesis and characterization. *Russian Chemical Reviews* **2016**, *85*, 280.

(S19) Putin, E.; Asadulaev, A.; Ivanenkov, Y.; Aladinskiy, V.; Sanchez-Lengeling, B.; Aspuru-Guzik, A.; Zhavoronkov, A. Reinforced Adversarial Neural Computer for de Novo Molecular Design. *Journal of Chemical Information and Modeling* **2018**, *58*, 1194–1204, PMID: 29762023.

(S20) Popova, M.; Isayev, O.; Tropsha, A. Deep reinforcement learning for de novo drug design. *Science Advances* **2018**, *4*, eaap7885.

(S21) Luong, K.-D.; Singh, A. Application of Transformers in Cheminformatics. *Journal of Chemical Information and Modeling* **2024**, *64*, 4392–4409, PMID: 38815246.

(S22) Park, H.; Li, Z.; Walsh, A. Has generative artificial intelligence solved inverse materials design? *Matter* **2024**, *7*, 2355–2367.

(S23) Fu, X.; Xie, T.; Rosen, A. S.; Jaakkola, T. S.; Smith, J. A. MOFDiff: Coarse-grained Diffusion for Metal-Organic Framework Design. The Twelfth International Conference on Learning Representations. 2024.

(S24) Schulman, J.; Wolski, F.; Dhariwal, P.; Radford, A.; Klimov, O. Proximal Policy Optimization Algorithms. *arXiv preprint arXiv:1707.06347* **2017**,

(S25) Haarnoja, T.; Zhou, A.; Abbeel, P.; Levine, S. Soft Actor-Critic: Off-Policy Maximum Entropy Deep Reinforcement Learning with a Stochastic Actor. *arXiv preprint arXiv:1801.01290* **2018**,

(S26) Park, H.; Majumdar, S.; Zhang, X.; Kim, J.; Smit, B. Inverse design of metal–organic frameworks for direct air capture of CO 2 via deep reinforcement learning. *Digital Discovery* **2024**, *3*, 728–741.

(S27) Majumdar, S.; Moosavi, S. M.; Jablonka, K. M.; Ongari, D.; Smit, B. Diversifying Databases of Metal Organic Frameworks for High-Throughput Computational Screening. *ACS Applied Materials & Interfaces* **2021**, *13*, 61004–61014, PMID: 34910455.

(S28) Li, B.; Wen, H.-M.; Cui, Y.; Zhou, W.; Qian, G.; Chen, B. Emerging Multifunctional Metal–Organic Framework Materials. *Advanced Materials* **2016**, *28*, 8819–8860.

(S29) Zhang, T.; Lin, W. Metal–organic frameworks for artificial photosynthesis and photocatalysis. *Chem. Soc. Rev.* **2014**, *43*, 5982–5993.

(S30) Furukawa, H.; Cordova, K. E.; O'Keeffe, M.; Yaghi, O. M. The Chemistry and Applications of Metal-Organic Frameworks. *Science* **2013**, *341*, 1230444.

(S31) Jiao, L.; Seow, J. Y. R.; Skinner, W. S.; Wang, Z. U.; Jiang, H.-L. Metal–organic frameworks: Structures and functional applications. *Materials Today* **2019**, *27*, 43–68.

(S32) Chen, L.; Xu, Q. Metal-Organic Framework Composites for Catalysis. *Matter* **2019**, *1*, 57–89.

(S33) He, Y.; Zhou, W.; Qian, G.; Chen, B. Methane storage in metal–organic frameworks. *Chem. Soc. Rev.* **2014**, *43*, 5657–5678.

(S34) Allendorf, M. D.; Stavila, V. Crystal engineering, structure-function relationships, and the future of metal-organic frameworks. *CrystEngComm* **2015**, *17*, 229 – 246, Cited by: 233; All Open Access, Green Open Access.

(S35) Safaei, M.; Foroughi, M. M.; Ebrahimpoor, N.; Jahani, S.; Omidi, A.; Khatami, M. A review on metal-organic frameworks: Synthesis and applications. *TrAC Trends in Analytical Chemistry* **2019**, *118*, 401–425.
